# Genome rearrangements induced by the stimulation of end-joining of DNA double strand breaks through multiple phosphorylation of MRE11 by the kinase PKB/AKT1

**DOI:** 10.1093/nar/gkaf468

**Published:** 2025-06-06

**Authors:** Josée Guirouilh-Barbat, Iman Litchy Boueya, Camille Gelot, Gaëlle Pennarun, Christine Granotier-Beckers, Elodie Dardillac, Wei Yu, Chloé Lescale, Emilie Rass, Olivier Ariste, Nicolas Siaud, Benjamin Renouf, Armel Millet, Nadine Puget, Pascale Bertrand, Pierre de la Grange, Erika Brunet, Ludovic Deriano, Bernard S Lopez

**Affiliations:** Université de Paris Cité, INSERM U1016, UMR 8104 CNRS, Institut Cochin, 24 rue du Faubourg St. Jacques, 75014 Paris, France; CNRS UMR 8200, Institut de Cancérologie Gustave-Roussy, Université Paris-Saclay, 114 Rue Edouard Vaillant, 94805 Villejuif, France; Université de Paris Cité, INSERM U1016, UMR 8104 CNRS, Institut Cochin, 24 rue du Faubourg St. Jacques, 75014 Paris, France; CNRS UMR 8200, Institut de Cancérologie Gustave-Roussy, Université Paris-Saclay, 114 Rue Edouard Vaillant, 94805 Villejuif, France; Université Paris-Saclay, INSERM, CEA, UMR Stabilité Génétique Cellules Souches et Radiations, F-92260 Fontenay-aux-Roses, France; Université Paris Cité, INSERM, CEA, UMR Stabilité Génétique Cellules Souches et Radiations, F-92260 Fontenay-aux-Roses, Frances; Université Paris-Saclay, INSERM, CEA, UMR Stabilité Génétique Cellules Souches et Radiations, F-92260 Fontenay-aux-Roses, France; Université Paris Cité, INSERM, CEA, UMR Stabilité Génétique Cellules Souches et Radiations, F-92260 Fontenay-aux-Roses, Frances; Université Paris-Saclay, INSERM, CEA, UMR Stabilité Génétique Cellules Souches et Radiations, F-92260 Fontenay-aux-Roses, France; Université Paris Cité, INSERM, CEA, UMR Stabilité Génétique Cellules Souches et Radiations, F-92260 Fontenay-aux-Roses, Frances; Université de Paris Cité, INSERM U1016, UMR 8104 CNRS, Institut Cochin, 24 rue du Faubourg St. Jacques, 75014 Paris, France; CNRS UMR 8200, Institut de Cancérologie Gustave-Roussy, Université Paris-Saclay, 114 Rue Edouard Vaillant, 94805 Villejuif, France; Institut Pasteur, Université Paris Cité, INSERM U1223, Équipe Labellisée Ligue Contre Le Cancer, Genome Integrity, Immunity and Cancer Unit, 75015 Paris, France; Institut Pasteur, Université Paris Cité, INSERM U1223, Équipe Labellisée Ligue Contre Le Cancer, Genome Integrity, Immunity and Cancer Unit, 75015 Paris, France; Université Paris-Saclay, INSERM, CEA, UMR Stabilité Génétique Cellules Souches et Radiations, F-92260 Fontenay-aux-Roses, France; Université Paris Cité, INSERM, CEA, UMR Stabilité Génétique Cellules Souches et Radiations, F-92260 Fontenay-aux-Roses, Frances; GenoSplice, 75014 Paris, France; CNRS UMR 8200, Institut de Cancérologie Gustave-Roussy, Université Paris-Saclay, 114 Rue Edouard Vaillant, 94805 Villejuif, France; Genome Dynamics in the Immune System Laboratory, Institut Imagine, INSERM, UMR 1163, Université Paris Descartes, Sorbonne Paris Cité, Equipe Labellisée Ligue Contre le Cancer, 75015 Paris, France; Genome Dynamics in the Immune System Laboratory, Institut Imagine, INSERM, UMR 1163, Université Paris Descartes, Sorbonne Paris Cité, Equipe Labellisée Ligue Contre le Cancer, 75015 Paris, France; MCD, Centre de Biologie Intégrative (CBI), CNRS, Université de Toulouse, UT3, 31062 Toulouse, France; Université Paris-Saclay, INSERM, CEA, UMR Stabilité Génétique Cellules Souches et Radiations, F-92260 Fontenay-aux-Roses, France; Université Paris Cité, INSERM, CEA, UMR Stabilité Génétique Cellules Souches et Radiations, F-92260 Fontenay-aux-Roses, Frances; GenoSplice, 75014 Paris, France; Genome Dynamics in the Immune System Laboratory, Institut Imagine, INSERM, UMR 1163, Université Paris Descartes, Sorbonne Paris Cité, Equipe Labellisée Ligue Contre le Cancer, 75015 Paris, France; Institut Pasteur, Université Paris Cité, INSERM U1223, Équipe Labellisée Ligue Contre Le Cancer, Genome Integrity, Immunity and Cancer Unit, 75015 Paris, France; Université de Paris Cité, INSERM U1016, UMR 8104 CNRS, Institut Cochin, 24 rue du Faubourg St. Jacques, 75014 Paris, France; CNRS UMR 8200, Institut de Cancérologie Gustave-Roussy, Université Paris-Saclay, 114 Rue Edouard Vaillant, 94805 Villejuif, France

## Abstract

Genetic instability is a major hazard threatening the fate of cells and ultimately of organisms. DNA double-strand break (DSB) is a highly toxic lesion, jeopardizing genome stability. Using cytogenetic and differential exome sequencing, we show here that upregulation of the kinase PKB/AKT1 leads to genomic rearrangements and chromosome fusions. By combining various approaches, at the genome scale and at precise loci, we show that PKB/AKT1 stimulates DSB end-joining, leading to inter- and intrachromosomal genomic rearrangements. The MRE11–RAD50–NBS1 (MRN) complex plays an essential role in the early steps of DSB signaling/repair. We show here that PKB/AKT1 favors the assembly of MRN, leading to the stimulation of DSB signaling via the MRE11/ATM axis. We identify MRE11 as a phosphorylation effector of PKB/AKT1 and reveal several sites whose phosphorylation is required for PKB-mediated stimulation of DSB end-joining and chromosome fusions. These data reveal that PKB/AKT1 actively promotes genetic instability by increasing the efficiency of DSB end-joining through MRE11 phosphorylation on these sites. These results highlight that not only a defect of DSB signaling/repair but also its stimulation, can lead to genome rearrangements and underline the importance of a precise regulation of the DNA damage response to maintain genome stability.

## Graphical Abstract

**Graphical Abstract:**
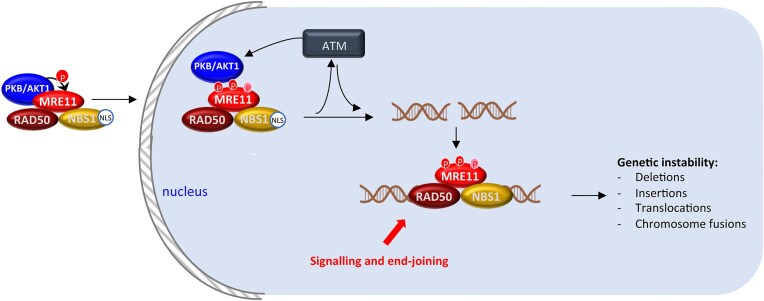

## Introduction

Genome instability leads to detrimental aftermaths such as developmental abnormalities, neurological disorders, cancer, premature aging, and death. It is thus essential to elucidate and dissect the molecular mechanisms controlling and driving rearrangements of the genome. DNA double-strand breaks (DSBs) are a prominent source of genomic rearrangements [1]. DSBs can result from exposure to exogenous genotoxic agents such as ionizing radiation (IR) as well as from endogenous sources such as genome replication accidents [2] or reactive oxygen species (ROS). DSBs can also be generated by controlled endogenous nucleases, to generate genetic diversity during physiological processes, including meiosis or the establishment of the immune repertoire. Therefore, controlling DSB repair is an essential issue for cells, in order to maintain genome stability while allowing genetic diversity, but avoiding genetic instability. Cells use two primary strategies for DSB repair, each containing subclasses of processes. The first strategy relies on homologous sequences with an intact DNA molecule and is referred to as homologous recombination (HR). The second strategy involves the ligation of two DNA double-strand ends (DSEs) without a requirement for sequence homology and is referred to as nonhomologous end joining (NHEJ). Both these processes are essential for genetic stability but can also generate genome rearrangements [1, 3, 4]. Any inappropriate choice of DSB repair mechanism or mis-combination of DSEs can severely alter genome organization. Notably, the joining of distant DSEs inevitably generates genomic rearrangements such as translocations, large deletions or inversions [1, 3, 5–17].

In response to genotoxic stresses, the DNA damage response (DDR) maintains genome stability through the coordination of a network of pathways, including DNA damage signaling and repair [18–20]. Remarkably, defects in the DDR network result in genetic instability, premature aging, and cancer predisposition [21, 22]. Sequencing of a panel of 52 426 tumors, including melanoma, hepatocellular carcinoma, and endometrial, gastroesophageal, ovarian, colorectal, biliary tract, bladder, breast, and pancreatic cancers, showed that 15%–34% of tumors exhibited mutations in DDR genes [23]. This observation highlights the importance of the DDR in cancer etiology.

Protein kinase B (PKB also named AKT1) is an oncogenic kinase [24], which is one of the most frequently upregulated oncogenes in diverse cancers [25]. Briefly, activation of the phosphatidylinositol 3-kinse (PI3K) leads to the phosphorylation of PIP_2_ lipids on the plasma membrane in PIP_3_ (phosphatidylinositol [3–5]-triphosphate), which favors the activation of the serine/threonine kinase PKB/AKT through its recruitment and interaction with PIP_3_ docking sites. A major antagonist is the phosphatase PTEN (phosphatase and tensin homolog), which dephosphorylates PIP_3_, resulting in the inhibition of the PKB signaling pathway [26, 27]. PKB endows resistance to apoptosis and stimulates cell proliferation but also inhibits several DDR pathways such as cell cycle checkpoints and HR [28–32]. In particular, the PKB axis is upregulated in a high percentage of sporadic breast and ovarian cancers [29, 33, 34]. As in parallel most mutations predisposing to familial breast or ovarian cancers affect genes controlling the DDR [35–37], this raises the question of whether PKB actually impacts genomic instability.

Upon DNA damage, PKB is activated by the three DDR-regulating PI-3 kinases ATM, ATR, and DNAPKcs [38–40] and co-localizes at DNA damages sites with the early DDR sensors γ-H2AX and ser1981 pATM [38, 41]. This suggests a putative apical function of PKB in the DDR, which remains to be elucidated.

The MRN complex (MRE11–RAD50–NBS1) is also recruited very early at sites of DSBs and is essential for the full activation of ATM signaling [42]. Reciprocally, MRE11 is phosphorylated by ATM and ATR after DNA damage [43]. MRE11 is also phosphorylated by cell cycle regulated kinases such as Cdc28/CDK1, CK2, or Plk1 and by ribosomal S6 kinase (S6K). Noteworthy, so far, all phosphorylations of MRE11 were reported to reduce MRE11 activity by decreasing its affinity for DNA and the recruitment of ATM at damaged sites. It is proposed that these phosphorylations control the turning off mechanism(s) of the MRN complex on DNA and DSB signaling.

Combining cytogenetic and differential exome sequencing, we show here that PKB upregulation induces genetic instability and chromosomal rearrangements. At a molecular level, we show that PKB phosphorylates MRE11 and that this potentiates DSB signaling. Indeed, PKB-mediated phosphorylation of MRE11 favors the assembly of the MRE11–RAD50–NBS1 (MRN) complex and enhances ATM signaling and EJ, *in fine* fostering intra- and interchromosomal genomic rearrangements (translocations, deletions, and inversions). This is, to our knowledge, the first report showing that MRE11 phosphorylations can stimulate its activity. Moreover, although PKB/AKT1 is hyperactivated in a wide variety of cancers, nothing was known about whether PKB itself generates genomic instability. Our data reveal the molecular mechanisms by which PKB actively promotes genetic instability.

The present data emphasize that the stimulation of DSB signaling and end-joining, through the MRN–ATM axis, can also promote genomic instability, underlying the importance of precise and balanced control of DDR equilibriums.

## Materials and methods

### Cells

GC92 cells [44] are derivates from the control simian virus 40 (SV40)-transformed fibroblast cell lines GM639 (male), a gift from R.J. Monnat Jr (University of Washington) and were originally obtained from the National Institute of General Medical Sciences Human Genetic Mutant Cell Repository (Camden, N.J.). They contain the CD4-3200bp substrate, which monitors the EJ-mediated deletion of a 3200 bp fragment or the EJ-mediated inversion of this fragment by expression of the membrane antigens CD4 and CD8, respectively. GCSH14 cells are derived from GC92 cells and contain the GFP-3200bp substrate, which monitors the same EJ-mediated deletion of a 3200 bp fragment by the expression of GFP [8, 45].

AID-DIvA (AID-AsiSI-ER-U20S) is a U2OS cell line (human osteosarcoma, female) in which DNA DSBs are induced at specific regions in the genome by the AsiSI endonuclease. AsiSI is sequestered in the cytoplasm, and after the addition of 4-hydroxy-tamoxifen (4OHT; 300 nM, Sigma–Aldrich, H7904) to the culture medium for 4 h, Asi-SI translocates into the nucleus and cleaves DNA. After 4OHT treatment, cells are washed three times in prewarmed PBS (Phosphate Buffered Saline) and further incubated with 500 μg/ml auxin (IAA) (Sigma–Aldrich, I5148) for 2 h to induce the degradation of AsiSI [46]. Then, the medium is refreshed, and the cells are cultured for 18–20 h before DNA collection.

All cell lines were cultured in DMEM supplemented with 10% FCS except for RPE-1-hTERT cells, which were cultured in DMEM-F12 supplemented with 10% FCS (Fetal Calf Serum). All cells were checked monthly for mycoplasma contamination by PCR (primers 5′ GGGAGCAAACAGGATTAGATACCCT 3′ and 5′ TGCACCATCTGTCACTCTGTTAACCTC 3′).

### Transfection

The meganuclease I-SceI, HA-PKB, FLAG-Mre11 WT, and the different FLAG-Mre11 mutants [Ser225Gly, Thr597Ala, Ser619Ala, and the triple mutant (TM)] were expressed by transient transfection with Jet-PEI following the manufacturer’s instructions (Ozyme, 101-40N) in all cell lines, except for DIvA cells, which were transfected using the Amaxa^™^ Cell line V kit (Lonza), according to the manufacturer’s protocol.

### Site-directed mutagenesis of Ser225, Thr597 and Ser619 of Mre11

Point mutants were generated by PCR-based methods using a QuikChange^®^ site-directed mutagenesis kit (Stratagene, 200 521) according to the manufacturer’s instructions. The primers used for mutagenesis were as follows (mutated codons are underlined):

- S225G-S: 5′-CAGAACAGGAGTAAACATGGAGGTACTAACTTCATTCCAGAAC-3′
- S225G-AS: 5′-GTTCTGGAATGAAGTTAGTACCTCCATGTTTACTCCTGTTCTG-3′
- T597A-S: 5′-GAGGAAGAGCAGACGCTGGTCTGGAGACTTCTACCCG-3′
- T597A-AS: 5′-CGGGTAGAAGTCTCCAGACCAGCGTCTGCTCTTCCTC-3′
- S619A-S: 5′-CTGCTGTGTCAGCATCTAGAAATATGGCTATTATAGATGCC-3′
- S619A-AS: 5′-GGCATCTATAATAGCCATATTTCTAGATGCTGACACAGCAG-3′

### Centromere-FISH on metaphase spreads

Centromere-fluorescent *in situ* hybridization (Centromere-FISH) was performed on metaphase spreads to quantify chromosome fusions. Seven days after transfection, cells were seeded at a density of 1 × 10^6^ cells per T25 flask. Twenty-four hours later, 0.1 μg/ml colcemid (Sigma–Aldrich) was added to the culture medium during the last 2 h. The cells were then harvested using trypsin and resuspended in pre-warmed hypotonic solution [0.0375 M KCl and 1/12 volume of human serum (Lonza) in H_2_O] for 20 min at 37°C. The cells were then fixed with ethanol/acetic acid (3V:1V) and stored overnight at 4°C. After washing in fixative solution, metaphase chromosome preparations were obtained by spreading the cells on SuperFrost microscope slides (VWR) using a cytogenetic drying system (Thermotron AdGenix). The slides were then rehydrated in PBS, fixed in 4% formaldehyde for 2 min at room temperature, washed in PBS, and dehydrated in graded ethanol solutions (50%, 70%, and 100%). Once the slides were completely dried, cells were incubated with 0.2 μM FAM(Fluorescein amidite)-labeled centromere probe (Eurogentec) diluted in hybridization solution (70% formamide, 10 mM Tris–HCl pH 7.2, 1% BSA, Bovine Serum Albumin) at 80°C for 3 min, followed by hybridization for 3 h and 30 min at room temperature in a humidified chamber. After hybridization, slides were then washed twice with buffer 1 (70% formamide, 10 mM Tris–HCl, pH 7.2) and were subsequently washed three times with buffer 2 (50 mM Tris–HCl pH 7.2, 150 mM NaCl and 0.05% Tween 20) and PBS. Chromosomes were counterstained with 1 μg/ml 4′, 6-diamidino-2-phenylindole (DAPI) (Sigma). After ethanol dehydration in graded ethanol solutions (50%, 70%, and 100%) and air drying, coverslips were mounted with Fluoromount-G (SouthernBiotech) and stored overnight at 4°C. Metaphases were imaged using a Zeiss Axioplan2 microscope (oil objective ×63) coupled with Metafer imaging software (MetaSystems). Fifty-one metaphases were analyzed per condition with ImageJ, and the experiment was repeated at least twice. The number of fusions per metaphase was calculated as follow: fusions per metaphase = dicentric + (tricentric *2) + sister fusion + chromatid-type fusion. Statistical analyses were performed using GraphPad Prism (GraphPad Software, Inc La Jolla, CA, USA). Histograms show the mean ± SEM.

### Exome sequencing

Exome capture was performed with the SureSelect V5 Mb All Exon Kit (Agilent Technologies) following standard protocols. Paired-end sequencing (100 bp) was conducted using the HiSeq2000 platform (Illumina) at the Fundamental Genomic Platform of Gustave Roussy Cancer Institute. Data analysis was performed by GenoSplice. In brief, quality control of fastq files was performed with fastqc (v11.2), and the coverage quality of the target regions was determined with GATK v3.5 (DepthOfCoverage). Sample reads were aligned to the human reference genome hg19 using BWA mem (0.7.12), and the read files were converted to bam format with samtools-1–1. Then, Picard-1.121 (SortSam) was used to sort the bam files by coordinates and mark duplicate fragments (MarkDuplicates). Next, the bam files were merged with Picard-1.121 (MergeSamFile) and indexed with samtools-1–1. Initial alignments were refined by local realignment using GATK-3.5 (RealignerTargetCreator, IndelRealigner). Last, base recalibration was performed on the bam files with GATK-3.5 (BaseRecalibrator, PrintReads). The single-nucleotide polymorphisms (SNPs) and insertions/deletions (INDELs) were called with GATK-3.5 (HaplotypeCaller). SVs were detected with five tools (lumpy v 0.2.13, delly 0.8.7, manta 1.6.0, rids 2.12.2 and svaba v1.1.3). VCF files generated by SV tools were merged by sample using SURVIVOR 1.0.7 with a maximum allowed distance of 1 kb; only calls supported by two callers were kept, and the event type was required to be identical. Pairwise comparisons between samples were performed with an in-house R script.

### Immunofluorescence

Immunofluorescence experiments were performed on cells grown on glass coverslips. Cells were fixed with 4% paraformaldehyde and were then permeabilized with 0.2% Triton X-100 for 15 min at RT. For immunofluorescence analysis of pATM (Ser1981) and pChk2 (Thr68), soluble proteins were extracted before fixation by incubating coverslips with extraction buffer [50 mM Tris–HCl (pH 7.4), 150 mM NaCl, 1% Triton X-100, and protease inhibitor cocktail (cOmplete Mini Protease Inhibitor, Roche, 5892970001] for 5 min on ice. After blocking in PBS containing 3% BSA and 0.05% Tween 20, immunostaining was performed using the following primary antibodies: mouse anti-53BP1 (1:200, Becton Dickinson, 612522), rabbit anti-53BP1 (1:100, Cell Signaling, 4937), mouse anti-HA (1:100, Covance, MMS-101R), rabbit anti-FLAG (1:300, Cell Signaling, 2368), mouse anti-pATM (1:250, Cell Signaling, 4526), and rabbit anti-pChk2 (1:500, Cell Signaling, 2661). After two washes with PBS containing 0.05% Tween 20, the coverslips were incubated for 30 min with Alexa Fluor 488- and/or 568-conjugated anti-mouse and anti-rabbit secondary antibodies (Life Technologies). All incubations were performed for 45 min at RT with antibodies diluted in PBS containing 3% BSA and 0.05% Tween 20. After two washes, the coverslips were mounted in mounting medium (Dako, S302380-2) supplemented with DAPI (Sigma–Aldrich). Images were acquired using a Leica SPE confocal laser scanning microscope or an Olympus BX63 microscope with a ×63 oil objective. Images were imported, processed and merged in ImageJ software.

### Detection of interchromosomal translocations in RPE-1 cells

RPE-1 cells were nucleofected with the expression plasmids for the relevant nuclease combination [47, 48]. To induce the t(1;19) translocation, cells were transfected with TALEN (TAL^LAM^), which cleaves a locus on Chr1, and TALEN (TAL^p84^), which cleaves a locus on Chr19 [10]. RPE-1 cells were eventually cotransfected with a PKB expression plasmid. The translocation frequency was calculated from a 96-well screen using small pools of cells and nested PCR to amplify translocation junctions. Frequencies were normalized to the number of viable cells 24 h after transfection. For statistical analyses, a *t* test was used for frequency comparisons.

### Linear amplification–mediated high-throughput genome-wide translocation sequencing

LAM-HTGTS was performed according to a published protocol [49]. In brief, DNA was purified and sonicated (Bioruptor, Diagenode) into 500–1000 bp fragments. LAM-PCR was performed using a biotinylated bait primer (CMV6, 5′-TGGTGATGCGGTTTTGGC-3′ for strategy 1 or CMV3, 5′-GTACGGTGGGAGGTCTATA-3′ for strategy 2) and 1 U Phusion polymerase (Thermo Fisher Scientific, F530L) with the following thermal cycling program: 1× (98°C for 120 s); 80× (95°C for 30 s, 58°C for 30 s, 72°C for 90 s), and 1× (72°C for 120 s). Biotinylated PCR fragments were incubated with MyOne streptavidin C1 beads (Invitrogen, 65001) with rotation for 4 h in buffer containing 1 M NaCl and 5 mM EDTA at room temperature. After washes with B&W buffer (1 M NaCl, 5 mM Tris–HCl, pH 7.4) and 0.5 mM EDTA (pH 8.0), on-bead ligation was performed using 2.5 μM bridge adapter, 1 mM hexamine cobalt chloride (Sigma–Aldrich, H7891), and 15 U T4 DNA ligase (Promega, M1804), 15% PEG-8000 (Sigma–Aldrich, P2139) with the following thermal cycling program: 25°C for 1 h, 22°C for 2 h, and 16°C O/N. After washing three times with B&W buffer, the on-bead ligation products were subjected to nested PCR using Phusion polymerase, a locus-specific primer (CMV3 for strategy 1 or T7, 5′-TAATACGACTCACTATAGGG-3′ for strategy 2) and adapter primers with the following thermal cycling program: 1× (95°C for 300 s), 15× (95°C for 60 s, 60°C for 30 s, and 72°C for 60 s), and 1× (72°C for 600 s). Restriction digestion was then performed with 5 U I-SceI (Thermo Fisher Scientific) for 2 h to remove uncleaved germline DNA. After purification using the QIAquick Gel Extraction Kit (QIAGEN, 28 704), recovered DNA was PCR-amplified using Illumina primers and Phusion polymerase with the following thermal cycling program: 1× (95°C for 180 s), 10× (95°C for 30 s, 60°C for 30 s, and 72°C for 60 s), and 1× (72°C for 360 s). The tagged PCR products were electrophoresed on a 1% agarose gel for size selection of DNA fragments of 500–1000 bp and purified using a QIAquick Gel Extraction Kit before loading into an Illumina MiSeq machine for paired-end 2 × 250 bp sequencing.

### LAM-HTGTS data analysis

(i) Read selection: the initial R1 reads containing the CMV3 (or T7) sequence (with an additional 20 nt of plasmid sequence at the 3′ end of the CMV3 or T7 sequence) were extracted using Cutadapt v2.8. A maximum error rate of 5% was allowed, and a minimum overlap of 40 nt was required for extraction. Subsequently, reads with an intact I-SceI site were discarded using Cutadapt v2.8 with the same error rate and overlap criteria. The read IDs were used to retrieve the corresponding matching pairs from the R2 fastq files. Finally, the R1 and R2 pairs were merged using flash2 to obtain longer reads. Further analyses were performed on the merged reads. (ii) Alignment: reads were mapped to the hg19 reference genome and CD4-3200bp plasmid sequence using bwa 0.7.17. The resulting alignment data were converted to BAM format and indexed using samtools. An in-house script was utilized to separate the reads mapped exclusively to the plasmid sequence from those mapped to both hg19 and the plasmid sequence. The BAM files were then converted to BED format using bedtools v2.27.1 (bam2bed). The mapping categories for each read were determined by overlapping the alignments in BED format with the regions in the plasmid sequence in BED format, employing bedtools (intersectbed). Reads within the same category were merged using bedtools (groupBy). A final table containing all samples was generated using an in-house script. (iii) Generation of Circos Plots: Circos plots were constructed using circosca tools. Links supported by fewer than three reads were discarded to ensure robustness. The number of reads was normalized using a logarithmic scale ranging from 0 to 25 000 reads.

### Inter- and intrachromosomal translocation assay in DIvA cells

AID-DIvA cells were treated as indicated, and DNA was then extracted from fresh cells using the DNeasy Kit (QIAGEN). The frequencies of translocations between different AsiSI sites in *MIS12* and *TRIM37* (chr17_5390209 and chr17_57184285, translocation TR 3), *LINC00217*, and *LYRM2* (chr6_135819337 and chr6_90348176, TR 6), *MIS12* and *LYRM2* (chr17_5390209 and chr6_90348176, TR 13), or *TRIM37* and *ASXL1* (chr17_57184285 and chr20_30946313, TR 1) were assessed by qPCR using the following primers:

- T3-F (5′GACTGGCATAAGCGTCTTCG-3′)
- T3-R (5′-TCTGAAGTCTGCGCTTTCCA-3′)
- T6-F (5′-GGAAGCCGCCCAGAATAAGA-3′)
- T6-R (5′-TCCATCTGTCCCTATCCCCAA-3′)
- T1-F (5′- CCGTCGGTCCTGTCTCAGTC-3′)
- T1-R (5′ AGTCGCCAAGTCTCGTATGC-3′).

The results were normalized using two control regions, both far from any AsiSI site and the γH2AX domain, using the following primers:

- Ctrl_chr1_82 844 750_Fw (5′-AGCACATGGGATTTTGCAGG-3′)
- Ctrl_chr1_82 844 992_Rev (5′-TTCCCTCCTTTGTGTCACCA-3′)
- Ctrl_chr17_9 784 962_Fw (5′-ACAGTGGGAGACAGAAGAGC-3′)
- Ctrl_chr17_9 785 135_Rev (5′-CTCCATCATCGCACCCTTTG-3′).

Normalized translocation frequencies were calculated using the DeltaDeltaCt method from [50].

### Analysis of DSB repair at the CD4-3200bp and GFP-3200bp intrachromosomal reporters

After transfection with the HA-I-SceI expression plasmid and incubation for 72 h, cells were collected with 50 mM EDTA diluted in PBS, pelleted, and fixed with 2% paraformaldehyde for 10 min. The cells were incubated for 10 min with 1 μl of an Alexa Fluor 647-conjugated anti-CD4 antibody (rat isotype, RM4-5, Invitrogen) and 1 μl of a PE-conjugated anti-CD8 antibody (rat isotype, 53-6.7, Pharmingen). The percentages of GFP-, CD4- and CD8-expressing cells were determined by FACS analysis using a BD Accuri C6 flow cytometer (Becton Dickinson). To eliminate variability due to the transfection efficiency, all values were normalized to those for control cells transfected with the I-SceI plasmid alone. Homogeneous expression of I-SceI among samples of each experiment is verified by western blot.

Where indicated, cells were treated with 2 μM PKB inhibitor IV (Calbiochem, 124 011), 10 μM triciribine (Calbiochem, 124 012), 10 μM DNA-PK inhibitor NU7026 (Selleck Chemicals, S2893), 10 μM ATM inhibitor KU55933 (Selleck Chemicals, S1092), or 10 μM mirin (Selleck chemicals, S8096) during the first 24 h after I-SceI transfection. Then, the medium was refreshed for the remaining 48 h of incubation before cells were collected for FACS analysis.

### Western blot analysis

Cells were lysed in buffer containing 50 mM Tris–HCl (pH 7.5), 20 mM NaCl, 1 mM MgCl_2_, and 0.1% SDS supplemented with cOmplete Mini Protease Inhibitor (Roche) and treated with 250 U of benzonase (Santa Cruz, sc202391) for 30 min. Proteins (30–40 μg) were denatured, separated on 9% SDS−PAGE gels, and transferred onto nitrocellulose membranes, which were incubated with the following specific antibodies: rabbit anti-PKB (1:1000, Cell Signaling, 9272), mouse anti-HA (1:1500, Covance, MMS-101), rabbit anti-p Ser824-KAP1 (1:5000, Bethyl Laboratories, A300767A), rabbit anti-actin (1:1000, Sigma–Aldrich, A2066), mouse anti-FLAG (1:1000, Sigma–Aldrich, F3165) and mouse anti-Vinculin (1:5000, Abcam, ab18058). Immunoreactivity was visualized using an enhanced chemiluminescence (ECL) detection kit (Pierce). Where indicated, 1 μM etoposide (sc-3512, Santa Cruz Biotechnology) was applied for 2 h before cells were collected.

### Cycloheximide treatment

When indicated, cells were treated with cycloheximide (50 μg/ml) for the indicated times prior collection.

### *In vitro* PKB kinase assay

The kinase activity of PKB and PKB-kd was measured with a nonradioactive IP kinase assay (Cell Signaling, 9840) following the manufacturer’s instructions.

### Proximity ligation assay

Cells grown on coverslips were fixed with 2% paraformaldehyde for 10 min, permeabilized, blocked and prepared as described above for immunostaining with the following primary antibody pairs: rabbit anti-MRE11 (1:100, Oncogene, PC388) and mouse anti-NBS1 (1:100, Thermo Fisher Scientific, MA1-23265), mouse anti-RAD50 (1:100, Abcam, ab89-100) and rabbit anti-PKB (1:500, Cell Signaling, 9272), rabbit anti-NBS1 (1:100, Oncogene, PC269) and mouse anti-HA (1:100, Covance, MMS101), and mouse anti-MRE11 (1:100, Abcam, ab214) and rabbit anti-PKB (1:500, Cell Signaling, 9272).

Proximity ligation assay (PLA) was performed using a Duolink In Situ Detection Kit (Sigma–Aldrich, DUO92001, DUO92005, DUO92008) according to the manufacturer’s protocol. Images were acquired with a Leica SPE confocal laser scanning microscope using a ×63 objective lens. Images were processed with ImageJ software.

### *In vitro* phosphorylation of recombinant Mre11 by recombinant PKB

*In vitro* phosphorylation was performed with recombinant Mre11 (Origen TP309414) and recombinant active PKB (Millipore 14-276) in the presence of 1× kinase buffer (Cell Signaling, 9802), 5 μM ATP (Cell Signaling, 9804), and 10 μCi ATP [γ-^32^P] (Perkin Elmer, BLU0021 100UC) at 30°C for 15 min. Reactions were stopped by adding Laemmli buffer with 4% β-mercaptoethanol and boiling for 5 min at 95°C. Samples were loaded on a 9% gel and transferred onto a nitrocellulose membrane, and radioactivity was visualized with Hyperfilm (Amersham).

### Coimmunoprecipitation

Cellular proteins were extracted on ice using 25 mM Tris–HCl (pH 7.5), 150 mM NaCl, 1 mM EDTA, 0.5% NP40 and cOmplete Mini Protease Inhibitor (Roche). Protein extracts were treated with DNase I (15 U/ml, Thermo Scientific, EN0521) for 30 min at RT. Extracts were precleared with Dynabeads (Life Technologies, 10004D) for 30 min at 4°C, and 300 μg of protein was then incubated with 1 μg of a mouse anti-FLAG antibody (M2, Sigma–Aldrich F3165) O/N at 4°C. Then, 25 μl of Dynabeads was added, and the mixture was incubated for 4 h at 4°C. The beads were subsequently washed three times with extraction buffer. Laemmli buffer (2×) with 4% β-mercaptoethanol was used to dissociate and denature the bead–antibody–protein complexes. Western blot analysis was performed to detect HA-PKB and FLAG-MRE11 using a mouse anti-HA antibody (1:1500, Covance, MMS101) and a mouse anti-FLAG antibody (1:1000, Sigma–Aldrich, F3165), respectively.

### Mass spectrometry

Samples were loaded and submitted to a SDS–PAGE gel migration. Gel slices corresponding to the mass of the proteins, i.e. 60 kDa for PKB/AKT1 and 80 kDa for MRE11 were cut. The gel slices were washed with a solution containing 50% of acetonitril and 50% of ammonium bicarbonate 25 mM, then dehydrated three times with a solution containing 100% acetonitrile. Each wash was carried out for 10 min at room temperature with shaking. The gel slice was dried using a Speed Vac. For trypsin digestion, the slices were pre-incubated with 20 μl of 11 μg/ml trypsin (Promega # V5111) at room temperature for 20 min. Afterward, 20 μl of 50 mM ammonium bicarbonate was added, and the gel slices were incubated overnight at 37°C.

Samples were centrifuged and the supernatants containing peptides were kept. The pellets were incubated with a solution containing 70:30 of acetonitrile and 5% formic acid for 20 min at 37°C. The supernatant is pooled with the first supernatant before being speed vacuum dried.

For MS and MS/MS, analyses were performed using a nanoHPLC (Agilent Technologies 1200) directly coupled to an ion-trap mass spectrometer (Bruker 6300 series). Briefly, peptides were resuspended in 10 μl of buffer containing 3% acetonitrile and 0.1% formic acid, and 2.5 μl was loaded and separated on a column with a 30 min gradient and a flow of 0.3 μl/min from 3% B (0.1% formic acid and 90% ACN) to 55% B.

The MS and MSMS spectra obtained were screened against the SWISS-PROT human database using a MASCOT search engine. The database searches were performed with methionine oxidation and serine, threonine, tyrosine phosphorylation, as variable modifications. Up to two missed cleavages was accepted.

### Statistical analysis

Statistical analyses were performed using GraphPad Prism 3.0 (GraphPad Software). Significant differences between experimental groups were analyzed by Kolmogorov–Smirnov or Kruskal–Wallis tests (*P < 0.05, **P < 0.01, ***P < 0.001, and ****P < 0.0001).

### Sequence data availability

Sequence data have been uploaded to - SRA database (NCBI) site for Exomes (PRJNA1045875) and HTGTS (PRJNA1045859).

## Results

### PKB induces genetic instability and chromosomal rearrangements in unchallenged human cells

**Figure 1.**
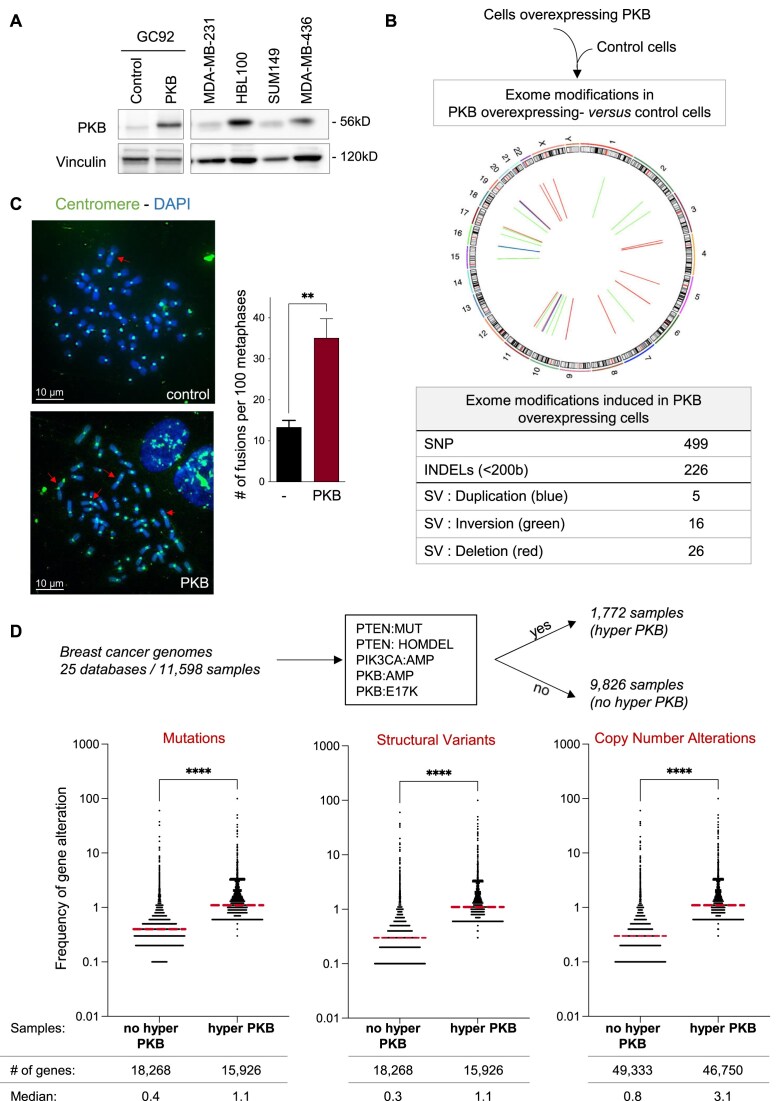
PKB induces spontaneous genetic instability and DSBs. (**A**) Western blot showing the expression of PKB in GC92 cells with control or PKB expression plasmids (left panel) versus spontaneous expression of endogenous PKB in different cell lines (right panel) (**B**) Differential exome sequencing. GC92 cells were infected with lentiviruses to guarantee stable expression of PKB for 2 weeks. Exomes of control cells and cells expressing PKB were sequenced, and bioinformatic analysis identified genome modifications that were present in the exome of PKB-expressing cells but absent in the exome of control cells. The Circos plot (middle panel) shows the localization of each SV detected. The table (lower panel) shows the numbers of SNPs, small INDELs (<200 bp), and SVs (i.e. duplications, inversions, or deletions). (**C**) Chromosomal fusions induced in metaphase spreads from GC92 cells transfected with PKB expression plasmid. Representative images of metaphase spreads stained with DAPI and a centromeric PNA probe are shown on the left side. Quantification of chromosome fusion events (means ± SEMs) are shown on the right panel. A minimum of 295 metaphases from four independent experiments were analyzed for each condition. Significant differences between experimental groups were analyzed by Kolmogorov–Smirnov test. (**D**) The analysis of all breast cancer databases in cBioportal (25 databases and 11 598 samples) reveals that PKB hyperactivation leads to an increased frequency of genomic alterations. Scatter plot represent the frequency of alteration in a panel of sequenced genes. Horizontal hatched (Red) line indicates the median. Significant differences between experimental groups were analyzed by Kolmogorov–Smirnov test.

First, we aimed at investigating the impact of PKB activation on interchromosomal rearrangements at the chromosome scale. Since PKB (AKT1) is upregulated in cancers [25], we overexpressed PKB in human SV40-immortalized fibroblasts (GC92) at a level that is comparable with that observed endogenously in a panel of breast cancer cells (Fig. 1A). Importantly, we observed that PKB up-regulation does not induce hyperproliferation nor a modification of the cell cycle distribution in our cellular system (Supplementary Fig. S1).

To evaluate the impact of PKB on genomic instability at the genome scale, we performed a differential exome sequencing analysis (Fig. 1B). After expression of PKB in GC92 cells, we identified genomic modifications in these cells compared to parental cells without PKB up-regulation (Fig. 1B). Although this approach restricts the analysis to the exome, it is a convenient method and is sufficient to reveal proneness to genetic instability at the genome scale [6]. PKB up-regulation induced the formation of 499 SNPs, 226 small INDELs, and 47 structural variants (SVs), of which 26 were deletions, 16 were inversions, and 5 were duplications (Fig. 1B).

Since the above method is not optimal to monitor interchromosomal exchanges, we analyzed the impact of PKB expression on chromosome fusion by a cytogenetic analysis. We observed that PKB up-regulation during one week increased the frequency of interchromosomal fusions by 3-fold (Fig. 1C).

Collectively, these data show that PKB expression induces genomic instability in unchallenged cells, i.e. in the absence of exogenous stress.

To investigate whether genomic instability is more important in tumors with PKB upregulation, we analyzed breast cancer genomic databases in cBioPortal and found that upregulation of PKB (through genomic alterations such as PI-3 kinase amplification, PTEN mutation or homozygous deletion, and PKB amplification or E17K mutation) is associated to a significant increase in the number of genomic alterations (mutations, SVs, and copy number alterations; Fig. 1D). These clinical data are at least in support of our experimental data.

Interchromosomal translocations, large deletions and inversions often result from the repair of DNA double strand breaks. Then we investigated whether PKB upregulation stimulates the occurence of genomic rearrangements at sites of DSBs.

### PKB promotes DSB-induced interchromosomal translocations

**Figure 2.**
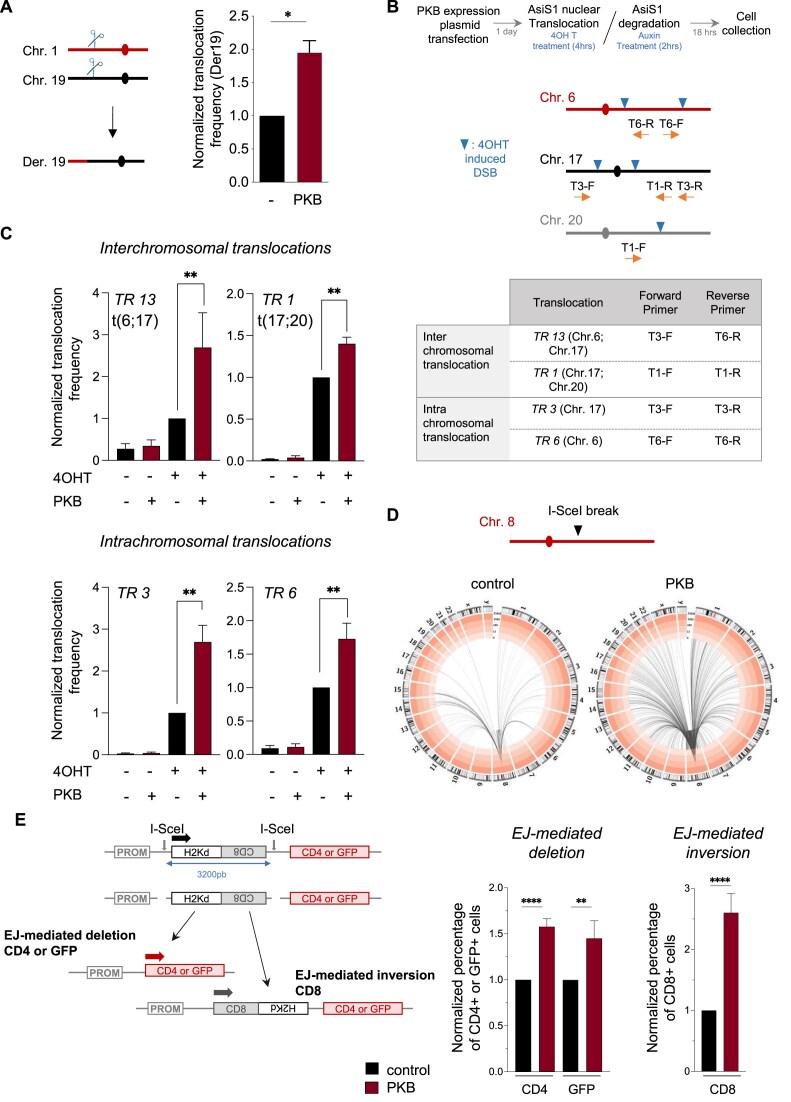
PKB stimulates DSB-induced inter- and intrachromosomal rearrangements. (**A**) Induction of the t(1;19) interchromosomal translocation in RPE-1 cells by sequence-specific nucleases: TAL^LAM^, which cleaves Chr1, and TALp84, which cleaves Chr19. The translocation frequency was calculated by PCR analysis of derivative chromosome Der19. The histograms show the means ± SEMs of three independent experiments. Significant differences between experimental groups were analyzed by Kolmogorov–Smirnov test. (**B** and **C**) Inter- and intrachromosomal translocations in AID DIvA cells. (B) The strategy used is shown in the upper panel. Breaks were induced by AsiSI (4OHT treatment) in AID DIvA cells (U2OS derivatives). Rejoining was detected by qPCR using specific primers as indicated in the scheme and in the table. (**C**) The histograms show the inter- (top panel) and intra- (lower panel) translocation frequencies (means ± SEMs, three independent experiments) before and after 4OHT + IAA treatment in AID DIvA cells transfected with the control or PKB expression plasmid. Significant differences between experimental groups were analyzed by Kolmogorov–Smirnov test. (**D**) LAM-HTGTS. A DSB was introduced in chromosome 8 of GC92 cells (in the CD4-3200bp reporter) by transient expression of I-SceI. After 72 h, the cells were collected and subjected to LAM-HTGTS. The Circos plots show the interchromosomal translocations (localization and frequency normalized to 25 000 total reads) detected after sequencing with primer CMV3 (see Supplementary Fig. S2). (**E**) Intrachromosomal rearrangements mediated by EJ. The CD4-3200bp and GFP-3200bp reporters (left panel). DNA breaks separated by 3200 bp were induced by I-SceI expression in the CD4-3200bp or GFP-3200bp reporter in GC92 and GCSH14 cells, respectively. Excision/deletion of the intervening fragment followed by EJ of the two distal DSEs leads to the expression of CD4/GFP. Inversion of the intervening fragment leads to the expression of the CD8 reporter. These events can be quantified by FACS in cells transfected with the control or PKB expression plasmid. The histograms (right panels) show the quantitative data (means ± SEMs) from 14 (EJ-mediated deletion, CD4+ cells, and CD4-3200bp reporter), 5 (EJ-mediated deletion, GFP + cells, and GFP-3200bp reporter), and 6 (EJ-mediated inversion, CD8 + cells, and CD4-3200bp reporter) independent experiments. Significant differences between experimental groups were analyzed by Kolmogorov–Smirnov test.

We first assessed the impact of PKB expression on an interchromosomal translocation induced by two targeted DSBs at specific loci on chromosomes 1 and 19 leading to the t(1;19) translocation. This translocation is reconstituted in cells with targeted endonuclease-mediated cleavage (here, mediated by TALENs) at specific loci [10]. Remarkably, PKB up-regulation increased the frequency of t(1;19) translocation induction by 2-fold in RPE-1 cells (Fig. 2A). This finding shows that PKB stimulates the joining of two DSBs located on two different chromosomes, leading to interchromosomal translocations.

To confirm the impact of PKB on interchromosomal rearrangements we used the AID-DIvA system (strategy shown in Fig. 2B, upper panel), which allows the quantification of interchromosomal translocations resulting from cleavage at different sites in the genome by the inducible endonuclease Asi-SI [51] (Fig. 2B). We found that PKB up-regulation significantly increased the frequency of interchromosomal translocations between chromosomes 6 and 17 (TR 13) and chromosomes 17 and 20 (TR 1) (Fig. 2C).

Then, to analyze the impact of PKB at the genome-wide scale, we used the Linear Amplification-Mediated High-Throughput Genome-wide Translocation Sequencing (LAM-HTGTS) approach [49, 52] adapted to an I-SceI site on chromosome 8 in the GC92 cell line. Consistent with the above data, PKB increased the frequency of interchromosomal translocations involving all chromosomes (Fig. 2D and Supplementary Fig. S2).

Collectively, these data show that PKB activation stimulates DSB-induced interchromosomal rearrangements.

### PKB stimulates intrachromosomal rearrangements induced by endonucleases

Differential exome sequencing analysis revealed that PKB also induced spontaneous intrachromosomal rearrangements such as deletions and inversions. To address the impact of PKB on intrachromosomal rearrangements induced by DSBs, we first used AID-DIvA cells (strategy shown in Fig. 2B, upper panel), exploiting specific primers to monitor intrachromosomal rearrangements (TR 3 and TR 6) (Fig. 2C). PKB also significantly increased the frequency of AsiSI-induced intrachromosomal translocations on chromosomes 6 (TR 6) and 17 (TR 3) (Fig. 2C).

**Figure 3.**
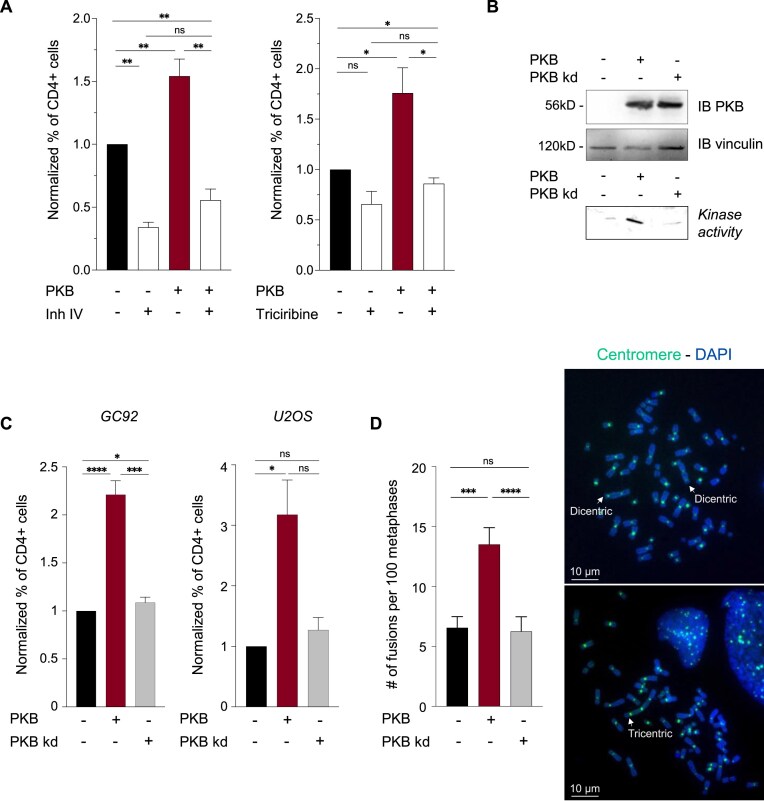
Stimulation of EJ-mediated deletion by PKB requires its kinase activity. (**A**) Frequency of EJ-mediated deletions (CD4+ cells) determined with the CD4-3200bp reporter in GC92 cells with or without transfection of the PKB expression plasmid and with or without PKB inhibitor IV (2 μM, left panel) or triciribine (10 μM, right panel) treatment for 24 h after I-SceI transfection. The histograms show the quantitative data (means ± SEMs) from 6 (PKB inh IV) and 4 (triciribine) independent experiments. Significant differences between experimental groups were analyzed by Kolmogorov–Smirnov test. (**B**) Top panel: Western blot showing the expression of PKB and PKB kd in cells transfected with the corresponding expression plasmids. Lower panel: measurement of PKB kinase activity in cells with or without transfection of PKB or its kinase-dead form (PKB kd). (**C**) Frequency of EJ-mediated deletions (CD4+ cells) determined with the CD4-3200bp reporter in GC92 and U2OS cells transfected with PKB or its kinase-dead form (PKB kd). The histograms show the quantitative data (means ± SEMs) for 10 and 4 independent experiments in GC92 and U2OS, respectively. Significant differences between experimental groups were analyzed by Kolmogorov–Smirnov test. (**D**) Chromosomal fusions induced in metaphase spreads from GC92 cells transfected with PKB or PKB kd expression plasmids. Quantification of chromosome fusion events (means ± SEMs) are shown on the right panel. Fifty-one metaphases were analyzed per condition with ImageJ, and the experiment was repeated twice. Significant differences between experimental groups were analyzed by Kruskal–Wallis test.

Second, we used intrachromosomal reporters allowing us to monitor deletions and inversions resulting from EJ of two distal DSBs induced in *cis* by the meganuclease I-SceI (Fig. 2E). We used two kinds of chromosomal substrates, CD4-3200bp and GFP-3200bp (Fig. 2E) [6, 45]. In these substrates, reporter genes are initially unexpressed because they are located too far from the promoter (GFP and CD4) or are in an inverted orientation with respect to the promoter (CD8). Two cut sites for the meganuclease I-SceI are inserted, leading to the excision of a 3200 bp internal fragment. The excision/deletion of this 3200 bp fragment followed by the EJ of the two distal ends leads to the expression of the CD4 or GFP reporter gene (CD4-3200bp or GFP-3200bp, respectively); the inversion and reinsertion of the 3200 bp fragment at the same locus leads to the expression of the CD8 reporter gene. These substrates have been extensively characterized and reproduce rearrangements (deletions and inversions) generated by EJ of distant ends [6, 8, 9, 44, 45, 53–55]. Here, PKB significantly stimulated the deletion of the 3200 bp intervening fragment associated with the joining of the 3200 bp distant DNA ends (EJ-mediated deletion), leading to the expression of CD4 or GFP, in GC92 human immortalized fibroblasts (Fig. 2E) and in U2OS cells (Fig. 3C). PKB expression also significantly stimulated the inversion of the 3200 bp intervening fragment (EJ-mediated inversion), as indicated by the expression of the CD8 reporter gene (Fig. 2E).

These data show that at two DSBs in *cis*, PKB expression promotes intrachromosomal rearrangements resulting from EJ of distant DSBs.

### Induction of DSB-induced genomic rearrangements by PKB requires its kinase activity

We then investigated whether PKB induces genomic rearrangements through its kinase activity. Both PKB inhibitors (PKB inhibitor IV, 2 μM and Triciribine, 10 μM) reduced the frequency of EJ-mediated deletion events in the absence of the exogenous PKB expression plasmid, suggesting a role for endogenous PKB in the basal frequency of such genomic rearrangements (Fig. 3A). In addition, treatment with PKB inhibitor IV or triciribine suppressed PKB-mediated stimulation of EJ-mediated deletion events (Fig. 3A).

In agreement with these data, the expression of a kinase-dead mutant of PKB (Fig. 3B) was unable to stimulate EJ-mediated deletion events in the SV40-transformed human fibroblast line GC92 (Fig. 3C). The requirement for the kinase activity of PKB was confirmed in U2OS cells harboring the same reporter (Fig. 3C). Together, these data show that the kinase activity of PKB is required for the stimulation of intrachromosomal rearrangements mediated by EJ of distant DSBs.

Finally, we showed that the overexpression of a kinase-dead mutant of PKB (PKB-kd) failed to stimulate the formation of interchromosomal fusions (Fig. 3D). These data at the chromosome scale are consistent with those above using intrachromosomal reporters and show that the kinase activity of PKB is required for chromosome rearrangements.

### Stimulation of EJ-mediated deletions by PKB involves ATM and MRE11

Using the CD4-3200bp reporter, we previously showed that EJ-mediated events can be promoted either by canonical nonhomologous end joining (C-NHEJ) or by alternative end joining (A-EJ) [6, 8, 9, 44, 45, 54, 55]. C-NHEJ catalyzes the direct ligation of the two 3′ overhangs generated by I-SceI cleavage in a conservative way. In contrast, A-EJ is characterized by deletions at the junction [1, 3, 44]. The relative contribution of each pathway (C-NHEJ versus A-EJ) can be measured by sequencing the EJ-mediated repair junctions on the CD4-3200bp substrate [6, 8, 9, 44, 45, 54, 55]. Using this strategy, we found that while PKB expression increased the global efficiency of EJ, it did not significantly impact the ratio C-NHEJ versus A-EJ (Supplementary Fig. S3 and Supplementary Table S1). This finding suggests that PKB stimulates EJ at a step preceding the choice between C-NHEJ and A-EJ, i.e. at an early step, such as the DSB signaling.

We previously reported that both ATM and MRN strongly regulate the efficiency of EJ-mediated deletion events measured with the CD4-3200bp reporter [44]. ATM, which is activated by the MRN complex, is primarily recruited to DSBs, triggering DSB signaling [56, 57]. Therefore, we investigated whether the signaling mediated by ATM and MRE11 promotes the stimulation of EJ-mediated deletion by PKB.

**Figure 4.**
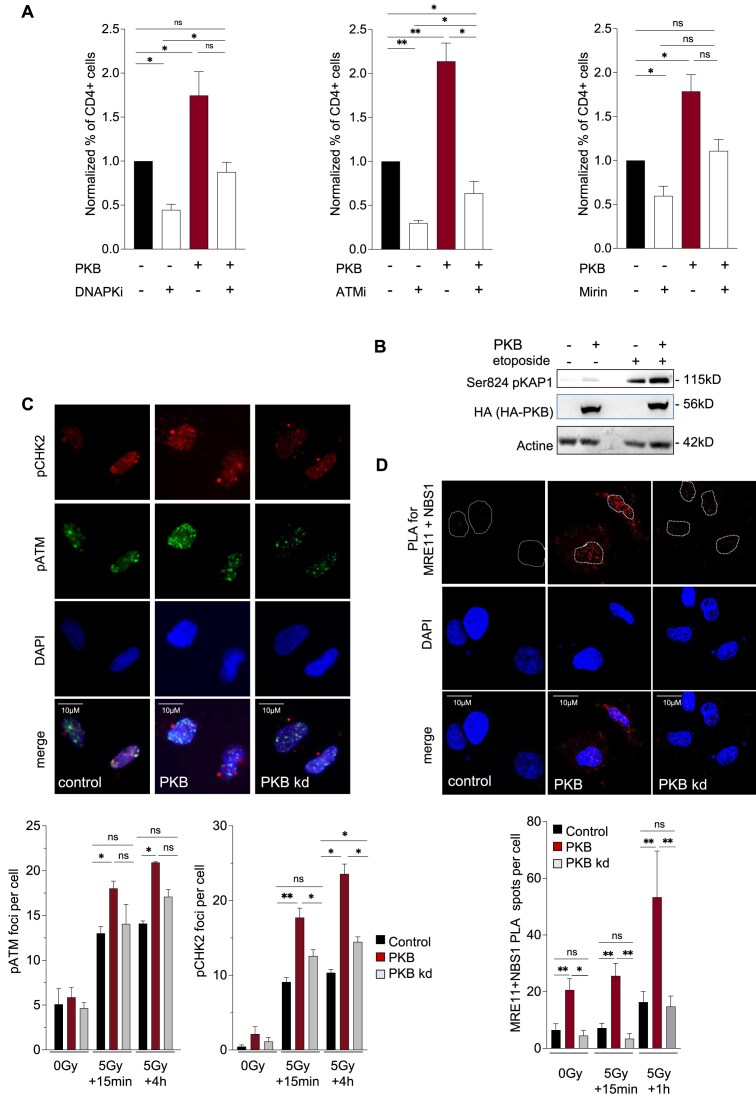
Stimulation of EJ-mediated deletion by PKB involves the activation of MRN/ATM signaling. (**A**) Frequency of EJ-mediated deletions (CD4+ cells) in GC92 cells with or without transfection of the PKB expression plasmid and with or without treatment with the DNA-PK inhibitor NU7026 (10 μM, left panel), ATM inhibitor KU55933 (10 μM, middle panel), or MRE11 inhibitor Mirin (10 μM, right panel) for 24 h after I-SceI transfection. The histograms show the quantitative data (means ± SEMs) from six (ATMi KU55933) and four (DNA PK inhibitor NU7026 and Mirin) independent experiments. Significant differences between experimental groups were analyzed by Kolmogorov–Smirnov test. (**B**) Western blot analysis of KAP1 phosphorylation at Ser824 in GC92 cells with or without transfection of PKB after etoposide treatment (1 μM, 2 h) where indicated. (**C**) pATM Ser1981 and pCHK2 Thr68 foci formation in GC92 cells expressing PKB or its kinase-dead form (PKB kd) with or without exposure to 5 Gy IR. Upper panel: representative images acquired 4 h after irradiation. Lower panel: quantitative data (means ± SEMs) from two to four independent experiments. Significant differences between experimental groups were analyzed by Kruskal–Wallis test. (**D**) PLA with probes targeting MRE11 and NBS1 in GC92 cells with or without exposure to 5 Gy IR. Upper panel: representative images acquired 1 h after irradiation. Lower panel: quantitative data (means ± SEMs) of puncta in three to nine independent experiments. Significant differences between experimental groups were analyzed by Kruskal–Wallis test.

First, exposure to a DNA-PK inhibitor (NU7026, 10 μM) abrogated the capacity of PKB to stimulate EJ-mediated deletion confirming that PKB acts on NHEJ (Fig. 4A). Moreover, an ATM inhibitor (KU55933, 10 μM) also abolished PKB-stimulated end-joining (Fig. 4A). Finally, Mirin (10 μM), which prevents MRN-dependent activation of ATM without affecting its protein kinase activity [58], also efficiently inhibited the stimulation of EJ-mediated deletion by PKB (Fig. 4A).

These data show that the ATM/MRN pathway is required for the PKB-dependent stimulation of DSB-induced genomic rearrangements.

### PKB enhances ATM signaling and MRN complex assembly

Considering the above data, we then tested whether PKB impacts ATM signaling after DNA damage induction. ATM phosphorylates KAP1 at serine 824 in response to DNA damage [59]. We found that PKB increased the phosphorylation of KAP1 by ATM in both untreated and etoposide-treated cells (2 h-treatment at 1 μM) (Fig. 4B).

We then investigated the recruitment of phosphorylated ATM to damaged DNA at different times after exposure to IR: 15 min after IR, corresponding to the early period of the immediate response to DNA damage, and 4 h after IR, corresponding to the period of sustained activation of ATM (Fig. 4C). Expression of wild-type PKB led to significant increase of IR-induced formation of Ser1981-pATM foci at both tested times after IR compared to that in control cells (Fig. 4C). In contrast, overexpression of a PKB kinase-dead mutant (PKB-kd) had almost no impact on IR-induced formation of Ser1981-pATM foci (Fig. 4C). The above data suggest that PKB stimulates DSB signaling mediated by ATM. To confirm these data, we analyzed the impact of PKB on foci formation of the phosphorylated CHK2, another canonical ATM effector, after IR. Expression of wild-type PKB significantly stimulated the IR-induced formation of Thr68-pCHK2 foci, while the stimulation observed upon expression of PKB kd was much weaker (Fig. 4C).

Collectively, these data show that PKB stimulates the ATM DNA damage signaling pathway.

ATM signaling is amplified by the MRN complex [56]. To investigate whether the formation and/or nuclear localization of the MRN complex is also enhanced by PKB, we performed PLAs to monitor the association between MRE11 and its partner NBS1.

Wild-type PKB stimulated the association of MRE11 with NBS1 spontaneously or after irradiation (5 Gy) (Fig. 4D), including in the nucleus of the cells where MRN exerts its effects. In addition, we observed an increase in NBS1 nuclear foci formation in PKB-expressing cells after IR (Supplementary Fig. S4). In contrast, expression of PKB kd failed to increase the association of MRE11 with NBS1 (Fig. 4D), showing that the formation of the MRE11/NBS1 complex depends on the kinase activity of PKB.

### PKB phosphorylates MRE11 *in vitro* and interacts with the MRN complex

The above data suggest that a member of the MRN complex could be a target for the kinase activity of PKB.

**Figure 5.**
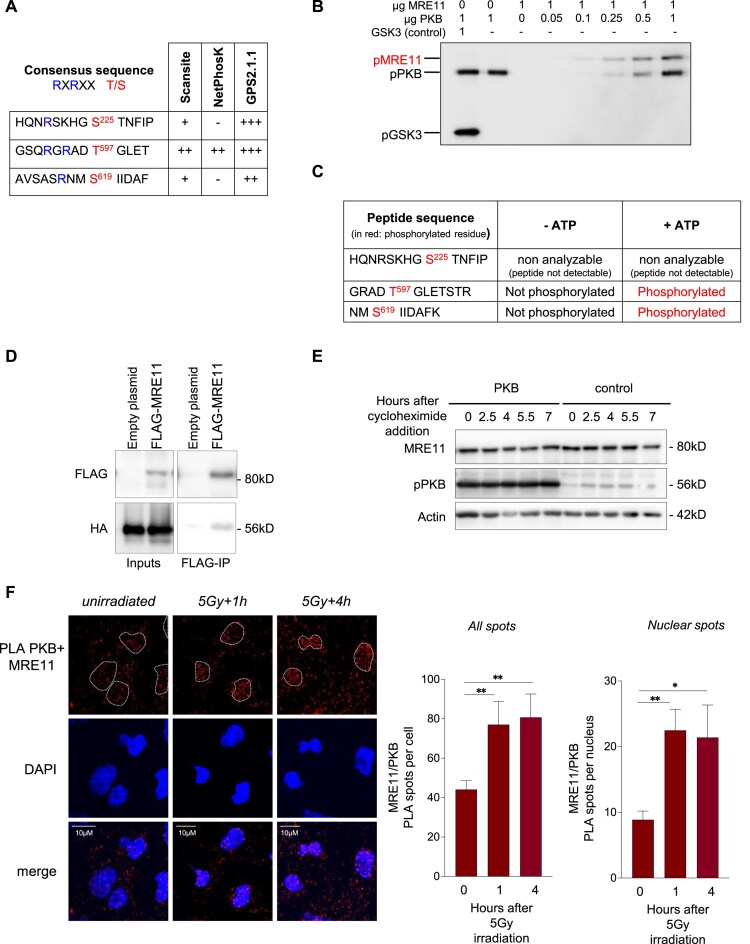
PKB phosphorylates MRE11 *in vitro* and interacts with MRE11 in cells. (**A**) *In silico* identification of MRE11 residues as potential targets of PKB kinase activity. (**B**) *In vitro* phosphorylation of MRE11 by PKB. The kinase activity of recombinant PKB toward recombinant MRE11 was evaluated by a radioactive assay in the presence of ATP [γ-^32^P]. In the first lane, pGSK3, a specific substrate of PKB, served as a positive control for PKB activity. In the second lane, no substrate was added. In the following lanes, 1 μg of MRE11 was incubated with increasing quantities of PKB. (**C**) Phosphorylated peptides of MRE11 identified by mass spectrometry. (**D**) HA-PKB coimmunoprecipitated with FLAG-MRE11. HA-PKB and FLAG-MRE11 were overexpressed in GC92 cells. Immunoprecipitation of FLAG-MRE11 was performed with an anti-FLAG antibody, and HA-PKB was detected in the coimmunoprecipitates by western blotting with an anti-HA antibody. (**E**) Western blot showing the level of MRE11 and PKB proteins in GC92 cells transfected with control or PKB expression plasmids and treated with the indicated times with cycloheximide (50 μg/ml). (**F**) PLA with probes targeting MRE11 and PKB in GC92 cells with or without exposure to 5 Gy IR. Left panel: representative images of unirradiated cells or cells irradiated with 5 Gy (after 1 h and 4 h). Right panel: quantitative data (means ± SEMs) of PLA puncta per cell (left histogram) or per nucleus (right histogram). Each value is presented as the average from 3 to 10 independent experiments. Significant differences between experimental groups were analyzed by Kruskal–Wallis test.

Therefore, we performed an *in silico* screen for potential PKB phosphorylation sites in the components of the MRN complex. No PKB phosphorylation sites were found in either RAD50 or NBS1, but three sites were identified in MRE11: serine 225, threonine 597, and serine 619 (Fig. 5A).

This finding prompted us to investigate the potential phosphorylation of MRE11 by PKB *in vitro* (Fig. 5B). PKB was found to undergo autophosphorylation as previously described [60] and to phosphorylate the positive control GSK3 peptide (first lane). Importantly, we found that PKB also phosphorylated MRE11 in a dose-dependent manner (Fig. 5B). A mass spectrometry (MS) analysis of the *in vitro* product confirmed the phosphorylation in presence of ATP of at least two sites (Thr597 and Ser619). The third site (Ser225) was present in a peptide that was not detectable by MS (Fig. 5C and Supplementary Table S2). Consistent with the above results, we showed by coimmunoprecipitation using Flag-MRE11 and HA- PKB that PKB and MRE11 physically interacted in cells (Fig. 5D) in the presence of DNase I, i.e. independent of the presence of potential DNA bridges. Phosphorylation of MRE11 by P70S6-kinase was reported to induce its degradation [61]. Therefore, we tested whether we also observed MRE11 degradation upon PKB overexpression. In our cell line, overexpression of PKB has no impact on MRE11 stability, following cycloheximide exposure (Fig. 5E). Since the MRN complex is mainly located in the nucleus, we then addressed the question of the sub-cellular localization of the interaction between PKB and MRE11.

We performed a PLA analysis to focus on the association between PKB and MRE11 in cells in response to DNA damage, i.e. after irradiation (5 Gy). The number of MRE11-PKB PLA puncta was significantly increased after IR (Fig. 5F); more specifically, the number of puncta in the nucleus was significantly increased (Fig. 5F), suggesting that IR promotes both the interaction between PKB and MRE11 and the localization of the PKB/MRE11 complex in the nucleus.

Notably, the PLA revealed that PKB was also localized very near the other components of the MRN complex, RAD50 and NBS1, in both the cytoplasm and nucleus of the cells (Supplementary Fig. S5).

Altogether, these data show that PKB interacts with the components of the MRN complex and that PKB phosphorylates MRE11, an event that might be part of the response to IR.

### PKB-mediated phosphorylation of Mre11 is required for the stimulation of EJ-mediated deletion

**Figure 6.**
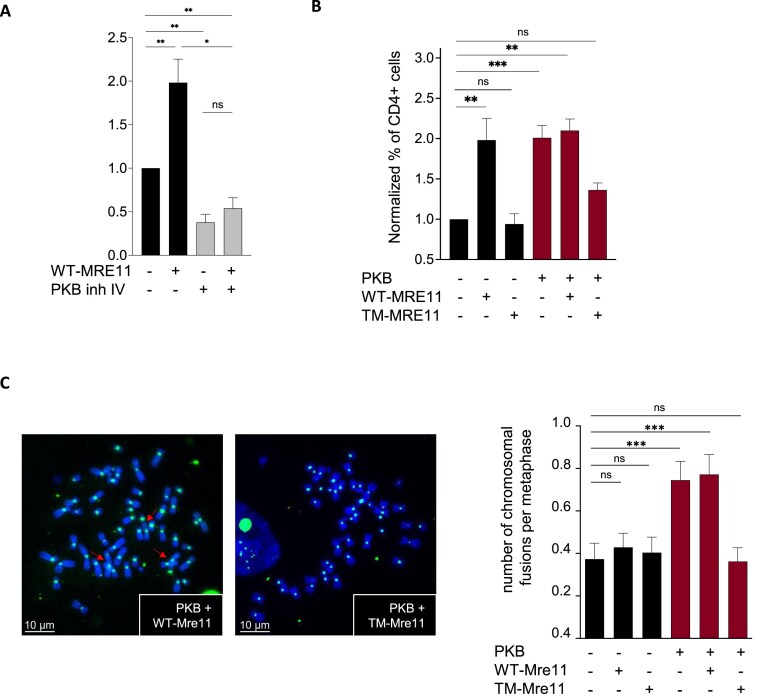
Phosphorylation of Mre11 by MRE11 is required for stimulation of genomic rearrangements. (**A**) Frequency of EJ-mediated deletions (CD4+ cells) in GC92 cells transfected with WT-MRE11 in presence of absence of a PKB inhibitor (inh IV, 2 μM). Histograms represent values from three to five individual experiments (means ± SEMs). Significant differences between experimental groups were analyzed by Kruskal–Wallis test. (**B**) Frequency of EJ-mediated deletions (CD4+ cells) in GC92 cells transfected with PKB and/or WT-MRE11 or TM-MRE11. Histograms represent values from three to five individual experiments (means ± SEMs). Significant differences between experimental groups were analyzed by Kruskal–Wallis test. (**C**) Chromosomal fusions in metaphase spreads from GC92 cells transfected with the control or PKB expression plasmid and/or the WT-MRE11 or TM-MRE11 expression plasmid. Quantification of fusion events (means ± SEMs) is shown on the Right panel. Representative images of metaphase spreads stained with DAPI and detected with a centromeric PNA probe are shown on the Left panel. A minimum of 75 metaphases were analyzed for each condition. Significant differences between experimental groups were analyzed by Kruskal–Wallis test.

Using the same CD4-3200bp reporter described in Fig. 2B, we previously showed that overexpression of MRE11 stimulates EJ-mediated deletion [44]. First, we show that inhibition of PKB (PKB inhibitor IV, 2 μM) abrogated the stimulation of EJ-mediated deletions (CD4+ cells) induced by MRE11 overexpression (Fig. 6A). This shows that active PKB is required for the stimulation of EJ-mediated deletions upon MRE11 overexpression. Then to test whether phosphorylation of MRE11 by PKB is required for this activity of MRE11, we determined the frequency of EJ-mediated deletions in cells overexpressing wild-type MRE11 (WT-MRE11) or the nonphosphorylatable MRE11 triple mutant (TM-MRE11), which contained mutations at the three sites identified by *in silico* analysis, i.e. Ser225, Thr597, and Ser619. First, up-regulation of either WT-MRE11 or PKB stimulated EJ-mediated deletion, as quantified with the CD4-3200bp reporter, with a similar efficiency (Fig. 6B). In addition, coexpression of PKB and WT-MRE11 did not further increase the frequency of EJ-mediated deletion, suggesting that PKB and MRE11 act in an epistatic manner (Fig. 6B). In contrast, overexpression of TM-MRE11 failed to significantly stimulate efficient EJ-mediated deletion and strongly decreased the stimulation resulting from PKB expression (Fig. 6B).

These data show that phosphorylation of MRE11 is required for the full stimulation of EJ-mediated deletion by PKB. To determine which of the three phosphorylation sites of Mre11 is necessary, we constructed forms of MRE11 with mutation of each of the three sites separately. In the absence of PKB, the expression of each single mutant failed to stimulate EJ-mediated deletion, in contrast with the expression of WT-MRE11 (Supplementary Fig. S6). Coexpression of PKB with either the Ser225 or Thr597 single mutant of MRE11 failed to increase EJ-mediated deletion. In the presence of the MRE11 Ser619 single mutant, PKB was still able to weakly stimulate EJ-mediated deletion, albeit with a more moderate effect than in the presence of wild-type MRE11. These data suggest that combined phosphorylation of at least Ser225 and Thr597 and, to a lesser extent, Ser619 in MRE11 might be required for the full stimulation of genetic rearrangements by PKB.

### PKB stimulates chromosome fusions through the stimulation of EJ via MRE11 phosphorylation

To investigate the impact of MRE11 phosphorylation by PKB at the chromosome level, we performed a cytogenetic analysis in cells overexpressing PKB and/or WT-MRE11 or TM-MRE11 (Fig. 6C). In the absence of PKB, the expression of either WT-MRE11 or TM-MRE11 did not impact the frequency of chromosome fusions, while coexpression of PKB and WT-MRE11 stimulated chromosome fusion (Fig. 6B). This pattern is consistent with the finding that PKB increased EJ efficiency due to the expression of phosphorylatable MRE11 (see Fig. 6A and B). In contrast, coexpression of PKB with TM-MRE11, which does not increase the EJ efficiency (see Fig. 6A and B), did not increase the frequency of chromosome fusions (Fig. 6B).

Taken together, these data reveal that the stimulation of EJ through PKB-mediated phosphorylation of MRE11 is required for the stimulation of chromosome fusions by PKB.

## Discussion

Here, we show that activation of EJ by PKB induces genomic rearrangements and chromosome fusions. Defects in the DDR network result in genomic instability [21, 22]. The present data show that, in a mirror effect, the stimulation of DSB signaling and repair via hyperstimulation of the ATM/CHK2 axis (herein found to be mediated by the kinase PKB) also promotes genomic instability. Because ATM and MRE11 are important factors for the maintenance of genome stability, the fact that their increased activation leads to genome rearrangement can appear paradoxical. However, the repair of DSBs by EJ is a double-edged sword. Indeed, on the one hand, it is essential for maintaining genome integrity and for resistance to IR, but on the other hand, it can generate genetic instability through EJ of distant DSBs, leading to translocations, deletions, inversions, and chromosome fusions [6, 8, 10–12, 14, 43]. DSB is a highly toxic lesion and unrepaired DSB lead to cell death. Therefore, activation of ATM and MRE11 leading to the stimulation of signaling and repair of DSBs should favor the combination of the survival of cells bearing DSBs associated with the stimulation of the EJ of distant DSBs that ineluctably leads to genetic rearrangements. This emphasizes the importance of precise and balanced control of these subtle equilibriums between DNA repair and cell death in the DDR. Moreover, our results reveal a novel role for PKB in DSB signaling and repair mediated through the phosphorylation of MRE11 and leading *in fine* to genomic rearrangements.

ATM phosphorylates MRE11 on S676 and S678, reducing the efficiency of HR repair [62]. These residues are also phosphorylated by other kinases depending on the type of DNA damage. Plk1 and CK2 phosphorylate S649 and S688 of MRE11, respectively, leading to the release of the MRN complex from DNA and the inactivation of both the ATM/CHK2 and ATR/CHK1 checkpoint pathways [63]. Here, we identified the first situation in which phosphorylation of MRE11 (herein by PKB) does not antagonize the MRE11/ATM axis but, in contrast, stimulates its activity in DSB signaling and repair, albeit at the cost of increased genetic instability. This is consistent with the high level of genomic instability observed in tumors characterized by PKB overexpression in cancer genome databases (cBioportal). Our data also reveal the molecular mechanisms by which PKB actively promotes genetic rearrangements and, in addition, give a molecular explanation for the radiation resistance of PKB-positive tumors [31, 64–66].

Several phosphorylation events have been described for MRE11 [67], and some have been characterized in numerous types of cancer, such as breast cancer, glioma, lung, and ovarian cancers (www.phosphosite.org). Notably, this list of MRE11 phosphorylation sites in cancers contains Thr597 and Ser619, which we identified in this study to be phosphorylated by the kinase PKB.

We show here that PKB promotes genomic rearrangements, leading to genetic instability. The impact of PKB on genomic rearrangements was shown herein in different types of cell lines, both cancerous and noncancerous (SV40-transformed fibroblasts, U2OS cells, and RPE-1 cells), with different read-outs (including natural genomic loci) and by analysis of breast cancer databases in cBioPortal. Regardless of the system used, all the data consistently led to the above conclusion, i.e. that PKB promotes genomic rearrangements and instability. We cannot exclude that, in addition, PKB overexpression increase the formation of endogenous DBSs. If this were the case this would create a potential synergy between the two processes to generate genetic instability. However, we show here that PKB stimulates genome rearrangements even upon the formation of independent exogenous DSBs (endonuclease), which constitutes by itself a genome instability process.

The relationships between PKB and DSB repair appear very complex and contradictory conclusions have been reported, notably for HR [28, 29, 68–72] and NHEJ. Indeed, reciprocal positive interactions between DNA-PK and PKB have been reported [38, 73, 74]. But, on another hand, PKB has been shown to phosphorylate the NHEJ component XLF, impeding DSB end joining (EJ) [75], which should thus confer sensitivity to IR. However, in contrast with these latter conclusions, PKB confers resistance to IR or chemotherapy [64–66, 76]. Here, we focused on the final outcome, i.e. the induction of genomic instability and characterized the mechanisms through which PKB promotes genomic rearrangements, associating two processes (see Graphical abstract): the stimulation of DSB signaling and EJ through the phosphorylation of MRE11; note that PKB interacts with MRE11 both in cytoplasm and nucleus and the kinase activity of PKB favors the assembly of MRE11 with NBS1. As NBS1 bears the NLS signal conveying the complex to the nucleus, PKB should indirectly favor the translocation of MRE11 into the nucleus. Since DSBs are the major toxic lesion induced by IR, the stimulation of the response to DSBs by PKB could account for the resistance of PKB-positive tumors to IR [31, 64–66].

Here we show that PKB expression also stimulates DSB signaling and EJ through phosphorylation of MRE11. Contradictory interactions between PKB and MRE11 have been reported. PTEN-deficient cells exhibit increased phosphorylation of MRE11 on the Thr597. This phosphorylation was found to induce the degradation of MRE11 [61]. However, this phosphorylation is not directly mediated by PKB but relies on P70S6-kinase [61]. Here, we show the direct phosphorylation of MRE11 by PKB, identify three phosphorylation sites in MRE11 but did not record increased degradation of MRE11. It is possible that the simultaneous phosphorylation on the three sites of MRE11 annihilates MRE11 degradation induced by the sole phosphorylation of the Thr597. In addition, in another study, PKB overactivation has been proposed to stabilize the MRE11 protein [77], and MRE11 was reported to promote ATM-mediated PKB phosphorylation in response to DNA DSBs [41], suggesting that PKB and MRE11 collaborate for an ATM signaling amplification loop.

Exposure of cells to genotoxic compounds such as doxorubicin, etoposide, and cisplatin or exposure to IR activates PKB [38, 78–80]. PKB is an effector of the membrane PI3-kinase, but it can also be activated by the other members of the PI-3 kinase family, which are central sensors of the DDR namely, DNA-PK, ATM, and ATR. In particular, DNA-PK directly phosphorylates PKB, increasing its activity [74]. PKB is also activated via ATM/ATR-dependent pathways, although no direct phosphorylation event was demonstrated [39, 40]. Additionally, PKB phosphorylation can be dependent on MRE11–ATM–RNF168 signaling [41]. Our data show that PKB stimulates MRN/ATM/CHK2 signaling, revealing a regulatory loop, at an early step of the signaling cascade. However, in contrast with the inhibition of the ATR/CHK1 signaling pathway [81], we show here that PKB stimulates the ATM/CHK2 cascade, leading to genomic instability. This finding highlights the importance of subtle and balanced control of the response to genotoxic stresses.

PKB is one of the most frequently upregulated oncogenes in diverse cancers [25], notably, in 40%–60% of sporadic breast and ovarian cancers [29, 33, 34]. PKB exerts pleiotropic effects, several of which account for its oncogenic power, such as stimulation of proliferation and inhibition of apoptosis. Since genetic instability is a hallmark of cancer cells [18, 82, 83], our data showing here that PKB promotes genomic instability reveal thus an additional oncogenic mechanism for PKB. Moreover, these data identify a new pharmacological target (MRE11) to optimize therapeutic strategies. Indeed, since many PKB-positive tumors appear to be resistant to radiation therapy, targeting NHEJ and, more particularly MRE11 should potentially sensitize cells to therapies based on genotoxic treatments, including radiotherapy. For example, PKB was found to be constitutively activated in 50%–80% of AML patients [84, 85]. Therefore, upon treatment that generate genetic rearrangements such as etoposide, the constitutive activation of the PKB signaling pathway should amplify the risks of genetic instability that might result in therapy-induced secondary leukemia. Our work identifies the EJ pathway, and more specifically MRE11 as possible targets to restrain such risks and to potentiate the efficiency of such treatment. Note that in one AML mice model, the combination of PI3K and DNA-PK inhibitors prolonged survival of treated mice [76]; one can suggest that MRE11 should represent an alluring alternative or additional target.

Our data reveal a novel mechanism generating genome instability: the stimulation of EJ that contributes to the generation of chromosomal rearrangements. In addition, our findings position PKB as a key apical regulator of the response to DNA DSBs. More generally, the present data show that, similar to defects in the DDR, the contrasting enhancement of DSB signaling and repair (here by PKB) also promotes genomic instability. These data underline the importance of precise and balanced control of DDR equilibriums.

## Supplementary Material

gkaf468_Supplemental_Files

## Data Availability

Sequence data have been uploaded to: SRA database (NCBI) site for Exomes (PRJNA1045875) and HTGTS (PRJNA1045859). Repair junctions on the CD4-3200bp reporter and Mass spectrometry data are provided in the supplementary files.
