# Supplementary material for: Genome rearrangements induced by the stimulation of end-joining of DNA double strand breaks through multiple phosphorylation of MRE11 by the kinase PKB/AKT1
Source: Nucleic Acids Res. 2025 Jun 6;53(11):gkaf468. doi: 10.1093/nar/gkaf468 (PMC12143594; doi:10.1093/nar/gkaf468)
Supplement: gkaf468_Supplemental_Files [file gkaf468_supplemental_files.zip › 3SUPP FIG NAR 2025-02-24.pdf]

A.

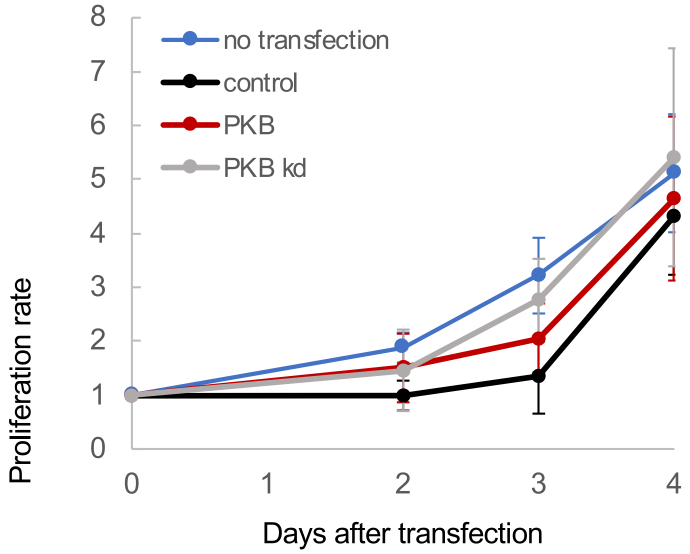

B.

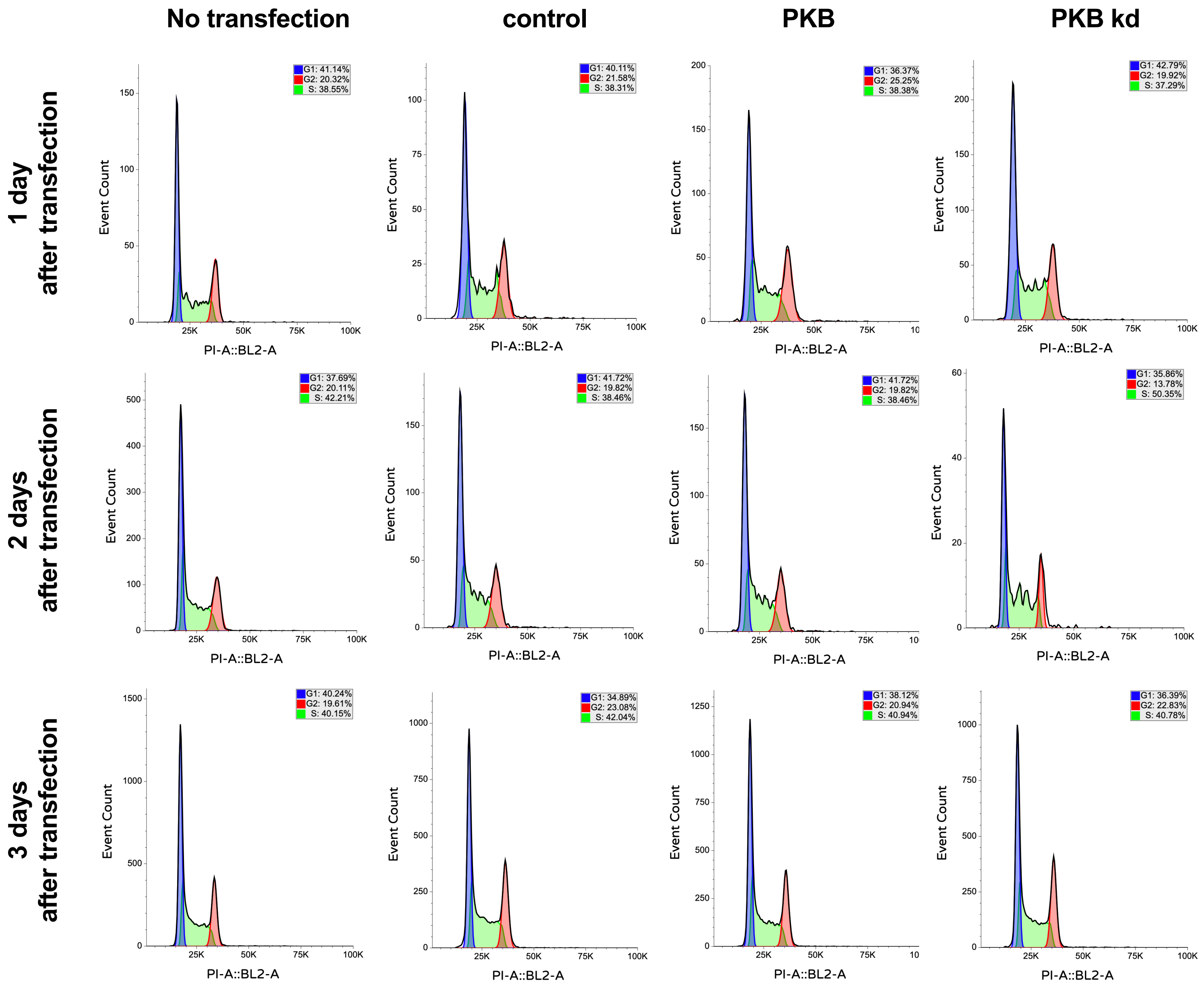

Supplemental Figure S1: PKB or PKB kd overexpression does not modify the proliferation rate (A), nor the cell cycle distribution (B) in GC92 cells.

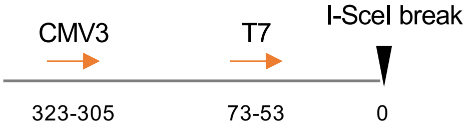

		# of reads with translocations	total # of reads	% of translocations among merged reads	fold increase in the frequency of translocations
Exp 1	control	37 740	973 066	2.99 %	
Sequencing with CMV3	PKB	12 962	431 748	4.37 %	1.46
Exp 2	control	2 867	47 829	5.99 %	
Sequencing with T7	PKB	7 115	69 057	10.30 %	1.72

Experiment 2
Sequencing with T7 primer:

Control PKB

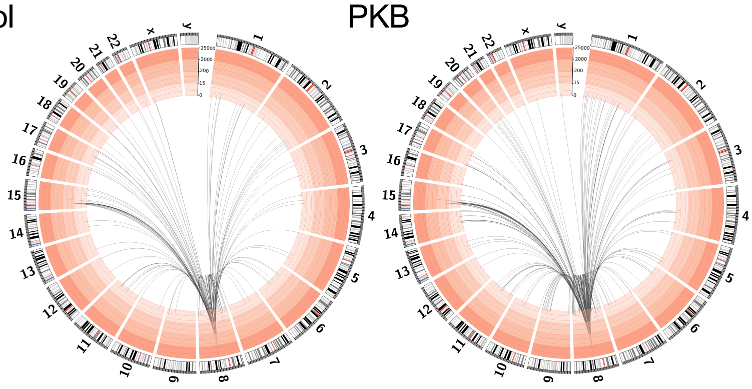

Supplemental Figure S2: Linear Amplification mediated High-Throughput Genome-wide Translocation Sequencing reveals a higher frequency of translocations in PKB expressing cells.

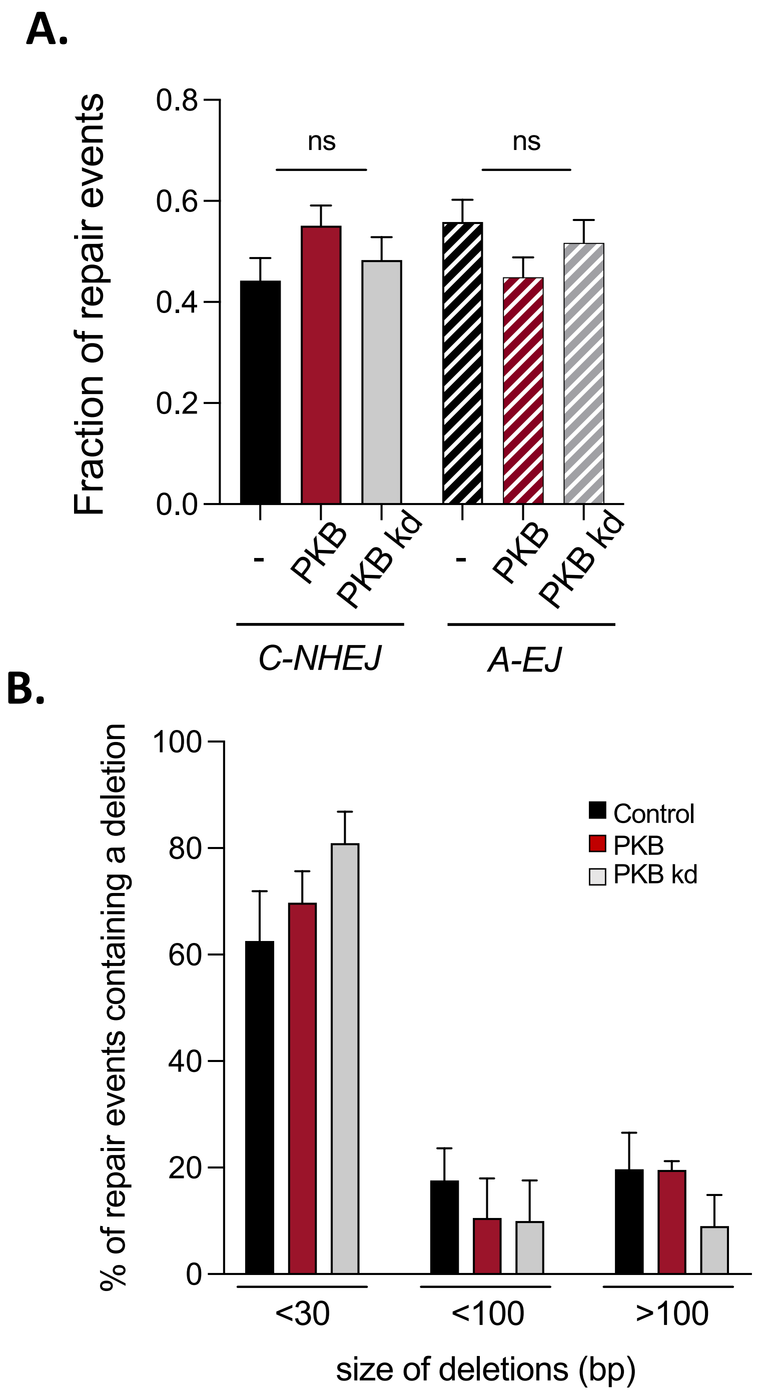

Supplemental Figure S3: A. Analysis of the C-NHEJ vs. A-EJ relative contribution with the CD4-3200bp reporter. Histograms represent the average of 2 experiments. **B.** Size distribution of deletions monitored with the CD4-3200bp reporter.

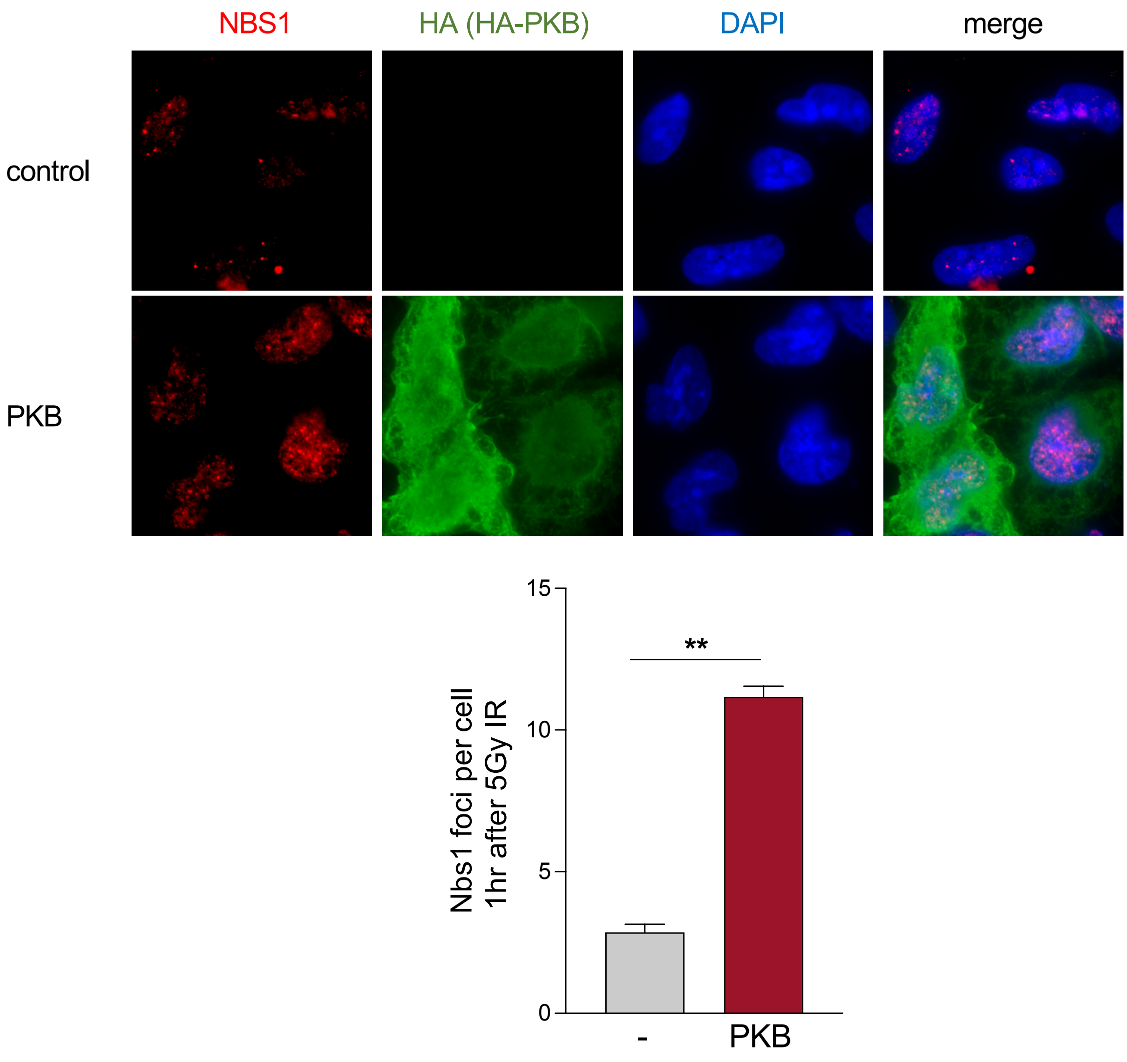

Supplemental Figure S4: PKB increases the number of NBS1 foci after irradiation

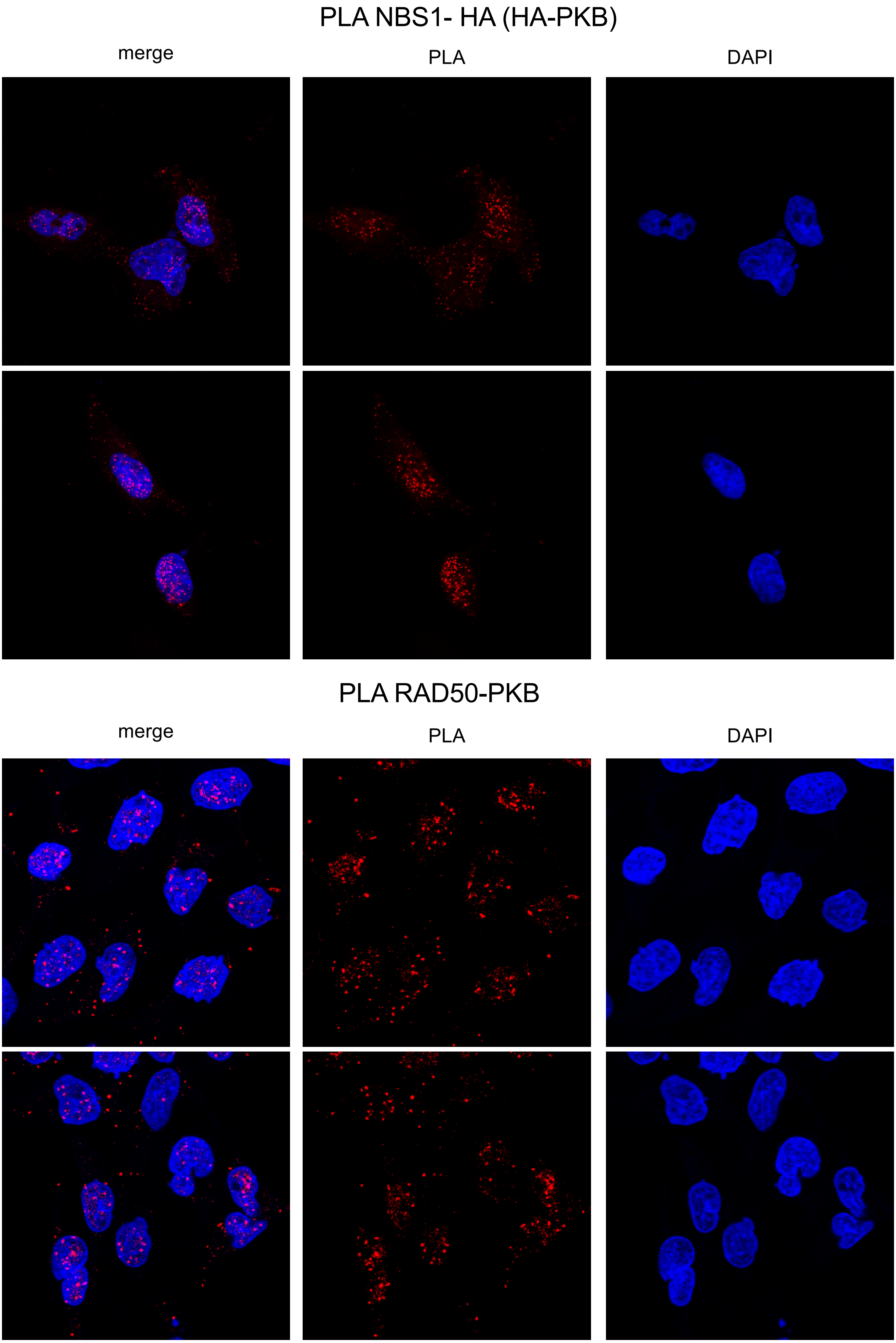

Supplemental Figure S5: Proximity Ligation Assay (PLA) with probes directed at NBS1and HA (for HA-PKB) or RAD50 and PKB in GC92 cells.

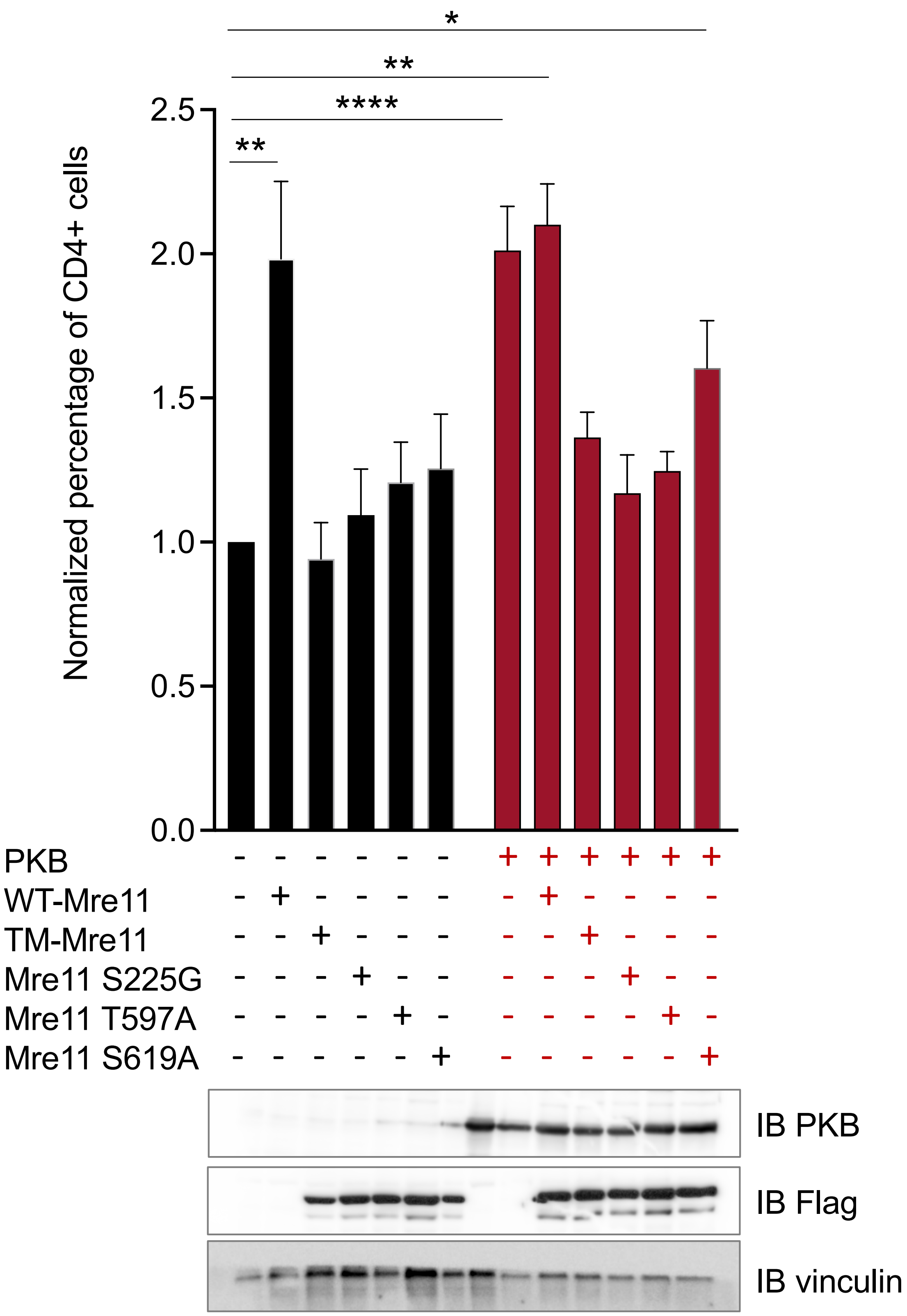

Supplemental Figure S6: the stimulation of DSB repair by PKB requires the phosphorylation on the three sites of Mre11: Ser225, Thr597 and to a lesser extent, Ser619.
